# Succession and persistence of microbial communities and antimicrobial resistance genes associated with International Space Station environmental surfaces

**DOI:** 10.1186/s40168-018-0585-2

**Published:** 2018-11-13

**Authors:** Nitin Kumar Singh, Jason M. Wood, Fathi Karouia, Kasthuri Venkateswaran

**Affiliations:** 1 Jet Propulsion Laboratory, California Institute of Technology, 4800 Oak Grove Dr, Pasadena, CA 91109 USA; 2 Space Bioscience Division, NASA Ames Research Center, Moffett Field, CA USA; 3 Department of Pharmaceutical Chemistry, University of California San Francisco, San Francisco, CA USA

**Keywords:** International Space Station, Metagenome, Functional metagenomics, Built environment, Propidium monoazide

## Abstract

**Background:**

The International Space Station (ISS) is an ideal test bed for studying the effects of microbial persistence and succession on a closed system during long space flight. Culture-based analyses, targeted gene-based amplicon sequencing (bacteriome, mycobiome, and resistome), and shotgun metagenomics approaches have previously been performed on ISS environmental sample sets using whole genome amplification (WGA). However, this is the first study reporting on the metagenomes sampled from ISS environmental surfaces without the use of WGA. Metagenome sequences generated from eight defined ISS environmental locations in three consecutive flights were analyzed to assess the succession and persistence of microbial communities, their antimicrobial resistance (AMR) profiles, and virulence properties. Metagenomic sequences were produced from the samples treated with propidium monoazide (PMA) to measure intact microorganisms.

**Results:**

The intact microbial communities detected in Flight 1 and Flight 2 samples were significantly more similar to each other than to Flight 3 samples. Among 318 microbial species detected, 46 species constituting 18 genera were common in all flight samples. Risk group or biosafety level 2 microorganisms that persisted among all three flights were *Acinetobacter baumannii*, *Haemophilus influenzae*, *Klebsiella pneumoniae*, *Salmonella enterica*, *Shigella sonnei*, *Staphylococcus aureus*, *Yersinia frederiksenii*, and *Aspergillus lentulus*. Even though *Rhodotorula* and *Pantoea* dominated the ISS microbiome, *Pantoea* exhibited succession and persistence. *K*. *pneumoniae* persisted in one location (US Node 1) of all three flights and might have spread to six out of the eight locations sampled on Flight 3. The AMR signatures associated with β-lactam, cationic antimicrobial peptide, and vancomycin were detected. Prominent virulence factors were cobalt-zinc-cadmium resistance and multidrug-resistance efflux pumps.

**Conclusions:**

There was an increase in AMR and virulence gene factors detected over the period sampled, and metagenome sequences of human pathogens persisted over time. Comparative analysis of the microbial compositions of ISS with Earth analogs revealed that the ISS environmental surfaces were different in microbial composition. Metagenomics coupled with PMA treatment would help future space missions to estimate problematic risk group microbial pathogens. Cataloging AMR/virulence characteristics, succession, accumulation, and persistence of microorganisms would facilitate the development of suitable countermeasures to reduce their presence in the closed built environment.

**Electronic supplementary material:**

The online version of this article (10.1186/s40168-018-0585-2) contains supplementary material, which is available to authorized users.

## Introduction

Places of human inhabitance have been a subject of study since *Homo sapiens* became a modern home-dwelling species in built environments. With advances in architecture for human inhabitance, dwelling places became controlled systems, including homes, offices, hospitals, and schools. Humans, being microbial fermenters, continuously interact with their environment at the microbial level. Most of the time, this interaction is balanced but can be highly tilted in a controlled system due to a continuous accumulation of one or more dominant microbes in the system. Long-term system studies have shown that the microbes of controlled environments were able to colonize the human gut, resulting in health concerns [1]. Another study by Lax et al. [2] shows that the microbial community of a new home reflects the microbial community of the occupants’ former house, suggesting a rapid colonization by the microbiota of the inhabitants. This suggests that the microbiota of the International Space Station (ISS) might be influenced by the arrival of a new crew.

The ISS is a prime example of a confined built environment, being more spatially isolated than any other human environment for its more than 18 years in continuous operation. The ISS, with its strictly controlled and highly monitored environmental systems (airflow under pressure, water circulation, and humidity), represents a unique opportunity to observe microbial community dynamics within a closed, engineered environment. The ISS is an ideal environment for studying the effects of microgravity and long-term space travel on colonizing microbes, their succession, and interaction dynamics with other microbes and astronauts [3].

Different aspects of space microbiology, utilizing traditional culture-based microbiological techniques, have been continuously studied on the ISS. These include assessments of the cultivable microbial burden [4], biofilm formation [5], and microbial effects on the structural integrity of ISS components [6] through bio-corrosive interaction with the constituent materials and metals [7]. Lessons learned from previous manned spacecraft missions and reports of various mechanical failure incidence due to microbial corrosion make it important to study and assess microbiomes of closed habitats [8, 9].

Even though healthy astronauts are visiting and inhabiting the ISS, microgravity has been shown to compromise the immune systems of its inhabitants [10]. Hence, microbes inhabiting the ISS can pose a serious health concern that must be monitored. Numerous studies have demonstrated that microbes that are exposed to microgravity become more resistant to antibiotics and more virulent [11–22]. For this reason, there has been a concerted effort to examine the microbiome of the ISS through numerous studies, such as, latent virus reactivation [11], antibiotic resistance properties [12, 13], and genomic characterization of ubiquitous [14–17] as well as potential pathogenic bacteria [18, 19] and fungi [20, 21]. Novel species have also been described from the ISS [22] for which virulence studies are warranted.

Characterization of emerging pathogens that could not be identified or those yet-to-be cultivated necessitates the importance of analyses of the microbiome utilizing various molecular techniques. Similarly, reports of microbial infection such as conjunctivitis, acute respiratory, and dental infections in MIR and space shuttle astronauts [23, 24] require the use of next-generation microbial detection techniques rather than simply relying on growth in culture media specified by spacefaring agencies. Pathogenic and virulent microbes, even though present in an environment, are outnumbered by native microbial inhabitants. In order to persist and succeed to infect, causative microbes require other factors like infectious dose concentration, dysfunctionality of the host (e.g., malnutrition and immunocompromised body), interaction with other symbiotic microbes that might provide nutrients, etc. These factors could not be simulated in a lab environment to culture all of the causative microorganisms. In this regard, the National Research Council recommended that National Aeronautics and Space Administration (NASA) utilize state-of-the-art molecular techniques to understand the baseline information about the ISS microbiome and its functional characteristics [3]. Capabilities to measure qualitative (gene sequencing) and quantitative (estimating gene copies) analyses were recently developed by NASA [25, 26], but these molecular techniques were not routinely used in the ISS due to non-availability of systems that could aid in sample collection, processing, or metagenomics library preparation targeting intact cells.

Analysis of intact cells is required as a first step to differentiate the dead cells from potentially viable microorganisms [27]. However, to correlate the metagenomics gene pool of intact cells with crew health requires additional functional characterizations such as virulence and pathogenetic analyses. Propidium monoazide (PMA) was used as a viability marker during this study to measure intact microorganisms. The PMA chemical is able to permeate into the compromised microbial cells and intercalate with nucleic acids [28–30]. Hence, PMA-intercalated DNA was no longer available for downstream molecular analyses such as quantitative polymerase chain reaction (qPCR) and shotgun sequencing, assisting in the estimation of gene copies and functional capabilities of intact microbial cells [27, 31].

Microbiome monitoring experiments conducted on the ISS were the microbial diversity analyses of the Kibo module [32], vacuum filter debris [33], HEPA (High Efficiency Particulate Air) filters [34, 35], ISS environmental surfaces [36], astronaut’s skin mycobiome [37], and Russian filter debris [38]. All of these studies were carried out using amplicon-targeted sequencing and were focused on microbial identification only; functional analyses were not performed. Amplicon sequencing facilitates the cataloging of microbial diversity, but when shotgun metagenomic approaches are employed on samples collected over time, microbial dynamics, antimicrobial resistance (AMR), virulence, and pathogenic characteristics of associated microorganisms can be predicted. Hence, metagenomics analyses would allow for the detection of etiological agents that might have the potential to cause health hazards to the ISS crew. Shotgun metagenome sequencing was used to analyze the ISS-HEPA debris, but whole genome amplification (WGA) protocols were necessary before generating metagenome sequences [35]. However, when WGA is employed, DNA from dominant microorganisms of a given sample will be enriched, whereas genetic materials of rare microbes will be unable to compete.

The present study is the first to generate shotgun metagenome sequences of intact microbial cells (PMA treatment) without WGA and to determine the functional capabilities of the ISS microbial community. This approach will help NASA to estimate succession, accumulation, and persistence of microorganisms, as well as AMR and virulence characteristics, and to design suitable countermeasures. The objectives of this study were to understand intact microorganisms associated with ISS environmental surfaces, their AMR and virulence profiles, and the succession of benign and pathogenic microorganisms in the samples collected from the ISS environment over a 12-month time period.

## Methods

### Description of sampled locations

Since the inception of the ISS, over 200 missions composed of periodic visits from international spacecraft for crew exchanges, resupply of food and other consumables, and many payloads for scientific investigations have occurred. The sampling on ISS surfaces performed for this study took place within the US on-orbit segments: Node 1, Node 2, and Node 3; US Laboratory Module; and Permanent Multipurpose Module (PMM). Based on the prioritization of surface locations and the efficiency of the crew procedure, the sampling plan was implemented as such: Node 3 (locations #1, #2, and #3), Node 1 (locations #4 and #5), PMM (location #6), US Laboratory (location #7), and Node 2 (locations #8 and control). A detailed description of various locations sampled is provided in (Additional file 1).

Location #1: Port panel of the cupola. The cupola is a small module devoted to the observation of operations outside the ISS, such as robotic activities, spacecraft approaches, and extravehicular activities (EVA). The cupola can accommodate two crewmembers simultaneously and is a popular spot for crewmembers during downtime. The panel (port side) on the way to the cupola was sampled. The panel is made of aluminum with a polyurethane topcoat (e.g., Aeroglaze A276 or BMS10-60).

Location #2: Forward side panel wall of the Waste and Hygiene Compartment (WHC). The (WHC), the space toilet, was the second toilet facility to arrive at the ISS. The wall surface on the back of the WHC was sampled. The panel is made of aluminum with a polyurethane topcoat (e.g., Aeroglaze A276 or BMS10-60).

Location #3: The foot platform of the Advanced Resistive Exercise Device (ARED). The (ARED) functions to maintain crew health in space. Crewmembers exercise daily on the ARED to maintain their preflight muscle, bone strength, and endurance. The foot platform of the ARED was sampled. The platform sampled during Flight 1 was made of gold-anodized aluminum. However, the platform sampled during Flight 2 and Flight 3 was subsequently covered with a black no-slip laminate (mineral grit embedded in an adhesive paper).

Location #4: Surface of the dining table. The original dining table sampled during Flight 1 and Flight 2 was a square surface (~ 0.6 m^2^). Even though the main function of the table was for dining, crewmembers also used the table for experimental work. As the number of permanent crewmembers increased over time, a new rectangular table (~ 1.25 m^2^) was installed in March 2016. The table is composed of a large and small leaf with latches in the middle and handrails on each side. The crew added tape, hook and loop fasteners, clips, and bungees to the table to hold their utensils and food in place. The material for the original dining table was polyimide whereas the new table materials were aluminum and stainless steel.

Location #5: Overhead-4- Zero-G Stowage Rack. Zero-G Stowage Racks (ZSRs; volume 1.21 m^3^) are fabric racks that are used onboard the ISS to provide stowage accommodations. The ZSR is a lightweight, on-orbit stowage restraint system. The ZSR comprises two elements: a collapsible shell and a fabric insert. The shell is an aluminum frame that provides a standardized interface to the insert. The front panel of the Overhead-4 ZSR was sampled. The white fabric surface material is based on Nomex. The content of the rack changed over time. During Flight 1, the rack contained the battery pantry, printer cartridges, office supplies (e.g., tape, Ziploc bags, and pens), dry vacuum supplies, cameras and cables, and trash bags. Whereas during Flight 2, the rack contained camera mounts, cables, blankets, Ziploc bags, and labels. Finally, during Flight 3, miscellaneous EVA camera parts were stowed in the rack.

Location #6: Port 1- Zero-G Stowage Rack and Port-2 Rack wall. The front surface of the port 1 ZSR was sampled during Flight 1 and Flight 2. The white fabric surface material is based on Nomex. During Flight 1, the rack contained clothes, crew preference items, office supplies, small tools (e.g., Leatherman and flashlight), ISS medical accessory kit. Whereas during Flight 2, the rack contained clothes, hygiene towels, cables, jumpers, caps, and food. During Flight 3, the PMM configuration changed, and Port-1 ZSR was not accessible for sampling. Therefore, the Port-2 panel, which is composed of aluminum honeycomb, was sampled instead. Near the sampling location, miscellaneous cables and accessories were present.

Location #7: Overhead-3 panel surface. The Materials Science Research Rack 1 (MSRR-1) is used for basic materials research in the microgravity environment of the ISS. MSRR-1 can accommodate and support diverse experiment modules. In this way, many material types, such as metals, alloys, polymers, semiconductors, ceramics, crystals, and glasses are studied to discover new applications for existing materials and new or improved materials. The Overhead-3 panel surface (LAB103) was sampled. The panel is made of aluminum with a polyurethane topcoat (e.g., Aeroglaze A276 or BMS10-60).

Location #8: Crew Quarters-2 Bump-out exterior aft wall. The Crew Quarters (CQ) is a permanent personal space for crewmembers to sleep and perform personal recreation and communication, as well as provide on-orbit stowage of personal belongings. The CQ was designed to provide 2.1 m^3^ of interior volume with an individual ventilation system, acoustical mitigation materials, radiation protection, light, and connections to provide power and internet for a laptop. The CQs provide visual, light, and acoustic isolation for the crewmember. The crew also uses the CQ for performing tasks such as donning/doffing clothing and some minimal personal hygiene. The structure of the CQ can be divided into three main areas: bump out, rack, and pop-up. The CQ-2 bump-out exterior aft wall was sampled during this study. The bump-out houses the ventilation system and is comprised of aluminum panels covered in acoustic absorption blankets which consists of a quilted configuration of Gore-Tex®, BISCO®, Durette felt, and Nomex™.

### Cleaning periodicity

Due to the accumulation of dust and debris, the crew is tasked with cleaning the ventilation system every 9 months. Additionally, weekly vacuum cleaning of the exterior mesh screens of the CQ takes place to reduce dust and debris build-up and provides crewmembers a clean sleeping environment. The present study requirements stated that there should be no cleaning at least 4 days prior to sampling. When the cleaning occurred during the weekends, it was carried out at the crew’s discretion without suggestions about the specific locations, therefore following the typical routine of activities on the ISS. The disinfectant wipes that are used in the ISS contain octyl decyl dimethyl ammonium chloride (0.0399%), dioctyl dimethyl ammonium chloride (0.01995%), didecyl dimethyl ammonium chloride (0.01995%), alkyl dimethyl benzyl ammonium chloride (50% C14, 40% C12, 10% C16), and dimethyl benzyl ammonium chloride (0.0532%). Unless otherwise stated above, the same eight locations were visited for each sampling event.

#### Sample collection and processing

Sample collection, processing, DNA extraction, and PMA treatment were carried out as described elsewhere [13, 27]. Briefly, sterile polyester wipes (23 cm × 23 cm; ITW Texwipe, Mahwah, NJ) were pre-moistened, folded two times, placed in a sterile Ziploc bag, and sent to the ISS for sample collection. Astronauts used these polyester wipes to collect 1-m^2^ samples from the same eight predefined locations during each sampling session. Samples were collected 7 days prior to the return on Flight 1, 9 days prior to the return on Flight 2, and 6 days prior to the return on Flight 3. Collected samples were stored at room temperature prior to return due to power restrictions on the ISS. Once returned to Earth, samples were stored at 4 °C until processing (within 24 h) in JPL facilities. During processing, the polyester wipes were aseptically removed from the Ziploc bags and transferred to sterile bottles containing 200 mL phosphate-buffered saline (PBS; pH 7.4). Bottles containing the wipes were vigorously shaken for 2 min to dislodge the sample from the polyester wipes. Each sample was concentrated using an InnovaPrep concentrating pipette (Drexel, MO) with 0.22 μm hollow fiber polysulfone tips (catalog #: CC08022) and PBS elution fluid. Two aliquots (1.5 mL each) were taken from concentrated samples, with one aliquot treated with PMA to assess intact cells. PMA solution (18.25 μL of 2 mM PMA) was added to each aliquot to bring its final concentration to 25 μM. Each aliquot was then incubated for 5 min at room temperature in the dark then exposed to the PMA LED activation system (Biotium, Hayward, CA) for 15 min. DNA extraction was performed using the Maxwell 16 System (Promega, Madison, WI) in accordance with the instructions provided by the manufacturer. Extracted DNA was eluted into 50 μL of sterile water and stored at − 20 °C until further analysis.

Control samples were included in all steps of the study for all three flight sessions. There was a field control, which was a wipe that was opened to the ISS environment but was not used for active sampling. A processing control, which was a no-template negative control with sterile molecular grade MilliQ water, was used during the DNA extraction steps. A reagent control that had no polyester wipe also served as a DNA extraction/PCR reagent control. In total, there were nine controls subjected to DNA extraction and subsequent DNA quantitation. None of the controls (both field wipes and reagents) and samples collected from location #6, whether PMA treated or untreated wipe samples, yielded DNA that could produce metagenomics libraries and hence did not proceed for shotgun Illumina sequencing (Additional file 1: Table S1). Metadata pertaining to the crewmember that performed sampling, the date of sample collection, and crew resupply vehicle information were already published [13].

### Shotgun metagenome sequencing

The initial DNA yield as measured by Qbit (Thermo Fisher Scientific Inc., USA) and metagenome library quantitation of all samples of the three flights, including controls, are given in Additional file 1: Table S1. The DNA yield from the nine control samples (three controls per flight) and samples from location #6 (all three flights) was below the detection limit (0.01 ng/μL). Subsequent metagenome libraries of these control and location #6 samples did not yield any shotgun metagenome sequences. However, amplicon-targeted sequencing showed the presence of microbial signatures for controls and location #6 samples (Checinska et al. 2018 submitted). DNA libraries for the remaining samples were prepared for shotgun metagenome sequencing using the Nextera DNA Library Preparation Kit from Illumina. The quality and fragment size of each library were assessed on the Bioanalyzer 2100 (Agilent). Separate adapters were added to the DNA from each library, normalized to 2 nM, pooled, denatured, and diluted to 1.8 pM according to the standard recommendations by Illumina. The HiSeq 2500 platform (Illumina) was used for sequencing, resulting in 100-bp paired-end reads.

### Metagenome sequence data processing

Paired-end 100 bp reads were processed with Trimmomatic [39] to trim adapter sequences and low-quality ends, with a minimum Phred score of 20 across the entire length of the read used as a quality cutoff. Reads shorter than 80 bp after trimming were discarded. All reads were normalized across samples as recommended by Nayfach and Pollard [40]. All 3 flight sessions, with 8 sampling locations and two treatments (PMA and non-PMA) accounted for 48 metagenomic samples. As all metagenomic sequencing library preparation reactions from location #6 failed, only 42 metagenomic samples were analyzed. High-quality filtered reads were clustered to respective taxonomic levels (domains through species) using the lowest common ancestor (LCA) algorithm provided by MEGAN6 [41] and normalized to do a semi-quantitative comparative analysis. ISS metagenome sequences were analyzed at individual flight level, i.e., Flight 1 to Flight 3 (temporal distribution), providing a holistic profile for the entire ISS. Metagenome sequences were also analyzed at the sample level, i.e., location #1 to location #8 (spatial distribution) for each of the flights to measure microbial dynamics (succession and persistence) for each particular location using statistical analyses detailed below. Microbial diversity analyses were carried out on normalized reads (~ 3.1 × 10^8^), and analyses were set to keep at least one unique read to minimize the loss of diversity in low depth samples or for unique reads. BLAST hits of ≥ 20 amino acids and ≥ 90% similarity were collected and used for taxonomic and functional assignment.

### Taxonomic and functional assignment

For lower downstream processing and visualization, the MEGAN6 [42] metagenomics toolkit was used. The NCBI taxonomy database [43], containing over 6.6 × 10^5^ reference sequences, and NCBI-NR protein sequence database, consisting of entries from GenPept, SwissProt, PIR, PDB, and RefSeq, were used to assign taxonomic features to reads by using DIAMOND [44] and the weighted LCA algorithm of MEGAN6 [41]. The identification of the reads to a taxon is not based on the *genes only*, but it is based on the comparison of the reads with the reference sequences deduced from the genomes of the curated NCBI taxonomy database [45]. Briefly, taxonomic and functional binning of the metagenomic reads is carried out using MEGAN [46], with the following settings: minScore = 50, maxExpected = 0.01, topPercent = 10, and minSupportPercent = 0.01. The resulting assignment of a taxon was presented in this manuscript. Functional analysis was carried out by mapping filtered DNA sequences against a reference database of all proteins within eggnog [47], SEED [48], and KEGG [49] databases. The search for translated DNA sequences was executed using DIAMOND, and hits that spanned ≥ 20 amino acids with ≥ 90% similarity were retained. In cases where one read matched these criteria against multiple proteins, only the protein or proteins (in the event of a tie) with the maximum bit score were considered. Pathways were analyzed by summing counts of KEGG orthologies for each pathway. Using different databases allowed a detailed view of reads defined by gene function consisting of a collection of biologically defined (i) subsystems, (ii) clusters of orthologous groups, and (iii) collection of metabolic pathways.

### Assignment of virulence

Out of the total microbial species reported from the ISS microbiome, risk group of Biosafety Level 2 (BSL-2) organisms was identified using the Bacterial and Fungal risk group database maintained by the American Biological Safety Association (https://my.absa.org/Riskgroups). Abundance profiles for the identified BSL-2 organisms were imported in Microsoft Excel to generate a 3D bar plot depicting the spatial and temporal distribution of these organisms.

### Clustering and statistical analysis

Clustering analysis of high-quality reads was performed using MEGAN6 to compute distances based on taxonomic and functional profiles obtained from NCBI taxonomy, SEED, and KEGG classification. The Bray-Curtis index was used to compute dissimilarity between samples. Calculated dissimilarities were then visualized with principal coordinate analysis (PCoA) plots. Species diversity was calculated using the Shannon-Weaver index (*H*) [50, 51] that considers both species richness and evenness in the community. Normalized read counts per taxon (from domain to species level) and read counts per function were exported as tables for further statistical analysis. Venn diagrams were produced using the R (http://www.r-project.org/) package venneuler [52] and a custom script (available from https://github.com/sandain/R/blob/master/vennplot.R) and VennDiagram (https://cran.r-project.org/web/packages/VennDiagram/). Analysis of similarities (ANOSIM) was carried out using the ANOSIM function from the R package vegan [53]. Mann-Whitney-Wilcoxon analyses were performed using the R function wilcox.test and a custom script (available from https://github.com/sandain/R/blob/master/mw.R). Multidimensional scaling (NMDS) was performed using the metaMDS function from the R package vegan [53] and a custom script (available from https://github.com/sandain/R/blob/master/mds.R).

In order to track the source of the microbial population of the ISS environmental surfaces examined in this study (2015 to 2016), metagenomes generated from various ISS-related samples were compared using multidimensional principal coordinate analysis (PCoA) [54]. The samples included for this analysis were ISS dust (collected during 1 day using a vacuum cleaner; 2012), ISS HEPA (particulates accumulated for > 40 months; 2011), Crew Resupply Service (CRS; that took cargo to the ISS in 2015 to 2016), and spacecraft assembly facility (SAF) cleanroom dust (2014) as well as SAF surfaces (2016) where cargo was assembled.

## Results

### Microbial diversity

Samples were collected from 3 flight sampling sessions on the ISS (8 samples for each flight and 24 samples in total). Each wipe was either treated with PMA or left untreated, resulting in an analysis of 48 samples and 9 controls. Among the 57 samples subjected for shotgun library preparation, only 42 samples (none from the controls and location #6) resulted in appropriate DNA yields (Additional file 1: Table S1). Approximately 7.3 × 10^6^ reads associated with microorganisms were generated after high quality trimming from PMA (21 samples) and non-PMA treated (21 samples) samples. All metagenomics reads were normalized across all samples, which yielded ~ 3.1 × 10^6^ in total, and ~ 7.4 × 10^6^ assigned to each sample, without affecting the taxonomic diversity. PMA-treated samples were the focus of this study as they represent the intact cells, and information about PMA-untreated samples were presented in supplementary datasets (Additional file 2: Table S2). Human-associated reads constituted ~ 1.75 × 10^6^ reads for non-PMA samples, whereas PMA treatment removed ~ 96% of human reads.

For all PMA-treated samples, at the domain level, the majority of the reads were assigned to bacteria (73.0%), followed by eukaryotes (26.9%) then viruses (0.1%), while archaeal signatures were not detected. For samples not treated with PMA, these reads were assigned to bacteria (76.0%), followed by eukaryotes (24.0%) and viruses (0.1%), but with a trace of archaeal signatures (Additional file 3: Figure S1). After metagenomic reads were normalized and analyzed for their relative abundance, it was evident that bacteria progressively increased from Flight 1 to Flight 3, whereas the trend was opposite for fungi. The proportional abundance of bacteria and fungi was similar in both PMA-treated and non-PMA-treated samples (Additional file 3: Figure S2). Viral signatures were found in Flight 1 and Flight 2 but not in Flight 3. *Archaea* were only found in low proportions in non-PMA-treated samples of Flight 1 and Flight 2. The top 25 species detected constituted ~ 80 to 92% of metagenomic reads (Additional file 3: Figure S3).

*Proteobacteria*, *Firmicutes*, *Ascomycota*, *Basidiomycota*, and *Actinobacteria* dominated the ISS microbiome profile at the phylum level. The percent abundance of *Proteobacteria* increased in Flight 3, while fungal populations of *Ascomycota* and *Basidiomycota* were reduced over the same time interval (Additional file 3: Figure S4A). At the class level, *Gammaproteobacteria*, *Eurotiomycetes*, *Alphaproteobacteria*, and *Bacilli* sequences were abundant. Sequences of *Gammaproteobacteria* were found to be more abundant in Flight 3 samples, whereas sequences of *Bacilli* were more abundant in Flight 2 samples. More than 98% of the total reads collected from PMA-treated samples from Flight 1 and 3 were assigned to *Enterobacterales*, *Bacillales*, *Rhizobiales*, and *Eurotiales* at the order level. In addition to these four orders, sequences associated with *Sphingomonadales* and *Pseudomonadales* were high in Flight 2 samples. At the family level, *Aspergillaceae* were present in all three flights and in all locations except location #7 on Flight 3. *Erwiniaceae* was the second most dominant family in all three flights in all locations of Flight 3. Taxa belonging to *Erwiniaceae* and *Staphylococcaceae* gradually increased from Flight 1 to Flight 3. Other common members were *Methylobacteriaceae* (Flight 1 and 2), *Staphylococcaceae* (Flight 2 and 3), and *Enterobacteriaceae* (Flight 1 and 3).

**Fig. 1:**
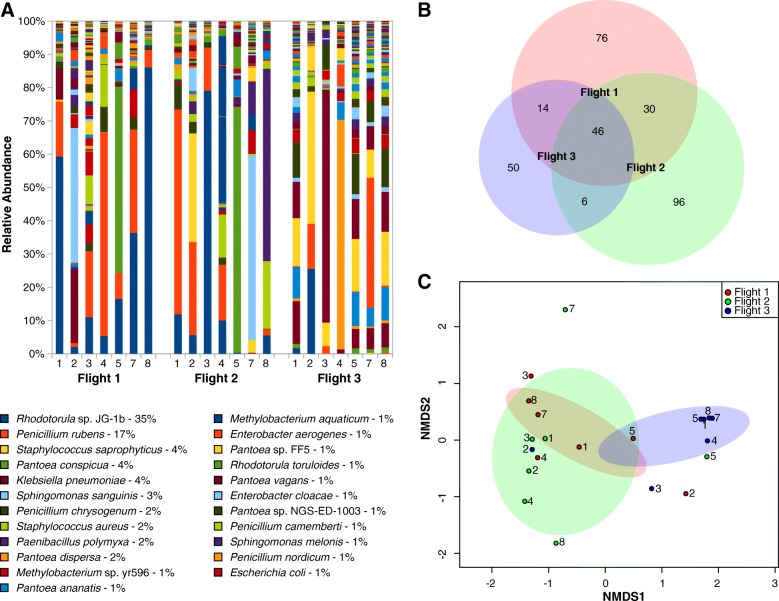
**a** Species-level abundance analysis based on normalized reads. **b** Venn diagram representing the common species between all three flights. **c** Species-based NMDS analysis representing various sampling sites in 2D ordinate as per the microbiome composition

Among the 115 genera identified, sequences of the members of the genera *Pantoea* (25 taxa) and *Penicillium* (12 taxa) were retrieved across all three flights. The compositional analysis showed a higher abundance of *Pantoea*, *Klebsiella*, *Staphylococcus*, and *Penicillium* in Flight 3 (Fig. 1a). In Flights 1 and 2, 6 and 9 different genera exhibited more than a million reads, respectively. A shift from fungi to bacteria was observed within a year of the first 2 sampling events (Additional file 3: Figure S4A). Among the 318 species identified, an increased abundance of *Klebsiella pneumoniae* and *Staphylococcus saprophyticus* sequences were noticed in Flight 3. The observed dominance of *Rhodotorula* sp. JG-1b and *Penicillium rubens* during the first 2 sampling events was replaced by the higher prevalence of various *Pantoea* species in Flight 3 (Fig. 1a).

All three flights shared several hierarchal levels of microorganisms (domain, 2 of 3; phyla, 9 of 9; class, 13 of 20; order, 20 of 56; family, 23 of 76). In particular, 31 out of 115 genera (Additional file 3: Figure S5B) and 46 of 318 intact microbial species (Fig. 1b) represented the core microbiome of all three flights. Among them, three species were dominant viz. *Rhodotorula* sp. JG-1b, *P. rubens*, and *S. saprophyticus*.

The analysis of similarity (ANOSIM) and Mann-Whitney-Wilcoxon statistics were used to compare microbial diversity from domain to species levels (Table 1). When microbiome constituents of all locations were pooled by flight, the taxa (domain to species level) significantly fluctuated in Flight 3 compared to the microbial communities sampled in Flights 1 and 2 (*p* = 0.01 to 0.0002; Table 1). However, the similarity in various microbiome components was statistically indistinguishable between Flight 1 and Flight 2 (*p* ≥ 0.01; Table 1).

**Table 1:** ANOSIM and Mann-Whitney-Wilcoxon analysis at domain to species taxon level

	ANOSIM					Mann-Whitney-Wilcoxon			
		F1P		F2P		F1P		F2P	
		*R*	*p*	*R*	*p*	*W*	*p*	*W*	*p*
Domain	F2P	− 0.03596	0.477			4	1		
	F3P	0.311	0.017	0.1293	0.065	5	1	5	1
Phylum	F2P	0.093929	0.2			34	0.5955		
	F3P	0.206	0.032	0.3003	0.024	41.5	0.9641	51	0.3762
Class	F2P	0.04179	0.26			208	0.8391		
	F3P	0.3829	0.016	0.4733	0.008	250	0.1781	256	0.1318
Order	F2P	0.05637	0.236			1115	0.9394		
	F3P	0.3693	0.017	0.5646	0.008	1388.5	0.02654	1438	0.01004
Family	F2P	− 0.02624	0.597			2778	0.6552		
	F3P	0.3751	0.013	0.4733	0.011	3359	0.004704	3334	0.00654
Genera	F2P	− 0.02624	0.572			6899	0.56		
	F3P	0.4393	0.01	0.4713	0.009	7249	0.1858	7074	0.3353
Species	F2P	− 0.03499	0.64			49,164	0.5258		
	F3P	0.45	0.013	0.4937	0.008	57,274	0.001452	58,476	0.000202

F1P Flight 1 PMA, F2P Flight 2 PMA, F3P Flight 3 PMA,

The relative abundance of phyla (*Ascomycota* and *Basidiomycota*), class (*Eurotiomycetes* and *Basidiomycota*), order (*Eurotiales* and *Sporidiobolales*), and family (*Aspergillaceae* and *Sporidiobolaceae*) were significantly different in Flight 3 compared to Flights 1 and 2. The difference in microbial genera between Flights 1 and 3 was statistically significant (*p =* 0.01) since the overlap in genus was limited to *Kosakonia*. Similarly, only 2 genera were common to Flights 2 and 3 (*Bacillus* and *Lactobacillus*) and statistically significant (*p* = 0.009). In addition to the core microbiome (46 species), 14 microbial species were shared between Flights 1 and 3 (*p =* 0.01) and only 6 species were common between Flights 2 and 3 (*p =* 0.008; Fig. 1b).

The Mann-Whitney-Wilcoxon statistics failed to detect any differences at the phylum- and class-level communities between all three flights, but significant differences were evident for Flight 3 at the order, family, and species levels (Table 1). The difference between the genera detected on Flights 1 and 3 was not significantly different (*p* ≥ 0.05). However, the species-level difference was robust and statistically significant between Flights 1 and 3 (*p* = 0.00145) as well as between Flights 2 and 3 (*p* = 0.00020).

NMDS analysis of the phyla demonstrated that the microbiome was similar among all locations of Flight 3, except location #2 (space toilet). However, when the distribution was analyzed by location, locations sampled during Flights 1 and 2 were different in their microbial composition compared to Flight 3. At the species level, most Flight 3 locations (5 out of 7) were grouped together separately from Flights 1 and 2, confirming that the microbiome composition of Flight 3 was dissimilar (Fig. 1c).

Location #5 (US Node 1, Zero-G Stowage Rack) of all three flights clustered together and was dominated by the members of family *Enterobacteriaceae*, genera *Pantoea*, and several species of *Pantoea*. In all levels of microbial taxonomy, Flight 3, location #2 (space toilet) microbial diversity was independent and not grouped within the microbiome associated with any other sampled ISS locations.

**Fig. 2:**
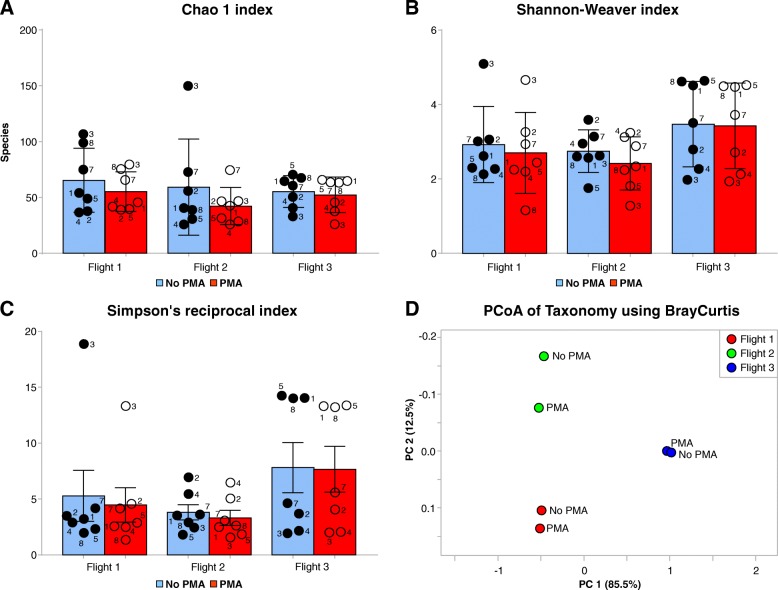
Species-level ordinate analysis of all three flight samples of ISS. Species-level diversity was determined using normalized reads. **a** Diversity analysis using the Chao1 estimator, **b** Shannon-Weaver index, and **c** Simpson’s reciprocal index. **d** Principal component analysis

All flight samples, PMA-treated and untreated, were subjected to various microbial diversity indices to mathematically measure the species diversity (alpha and beta diversity) which includes (i) Chao1 (Fig. 2a), (ii) Shannon diversity index (*H*; Fig. 2b), (iii) Simpson’s diversity index (Fig. 2c), and (iv) principal coordinate analysis (PCoA; Fig. 2d). Species diversity increased between Flights 1 and 2 but was reduced in Flight 3. It was also evident that diversity was reduced in all PMA-treated samples. The Chao1 alpha diversity index showed that there was a decrease in the species diversity of PMA-treated samples. The Shannon-Weaver index *H* value varies from 1.0 to 4.5 (for PMA-treated samples) from Flight 1 to Flight 3, which is indicative of a compositional shift in the metagenomics community. In addition, a higher *H* value indicates the distributive evenness of species in Flight 3 sampling, but it also represents the collective convergence of all sampling locations. When individual sampling locations of each flight were taken into account (e.g., sampling locations 3, 5, and 8), a fluctuation was seen in the value of *H* relative to the flight, demonstrating the highly dynamic nature of the intact microbial communities of these sampling locations. Microbial diversity analysis was also carried out through Simpson’s index, which is a measure of diversity based on richness and evenness of species in a sample. The Simpson index confirmed the Shannon diversity results as Flight 3 scored higher in both analyses. When microbial profiles were analyzed by flight, it was evident from the two-dimensional PCoA analyses that each flight represents a distinct microbial profile (Fig. 2d).

### Sequence detection of cultivated microorganisms from the metagenomic dataset

**Fig. 3:**
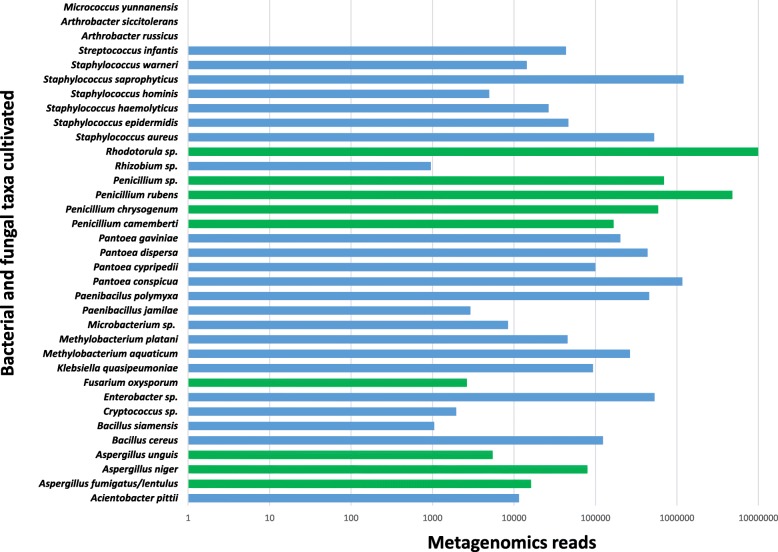
Abundance of metagenomics reads retrieved in PMA-treated samples that showed the presence of cultured bacteria (blue) and fungi (green). Three microbial species observed in culture analyses of PMA-treated samples were not observed in the metagenomic data set

Metagenomics data were mined for the presence of genetic signatures associated with the organisms cultured from the same samples. The reference database used in this study contained reference signatures for all 35 cultured isolates identified at the species level. Of the 35 cultured species from these samples [55], metagenomics sequences of 32 isolates were found in PMA-treated samples (Fig. 3). In total, the metagenomics pipeline employed during this study retrieved reads pertaining to 23 bacterial and 9 fungal species of cultivated microorganisms. Metagenome reads associated with the cultivable fungal species such as *Aspergillus niger*, *Penicillium camemberti*, *Penicillium chrysogenum*, *Penicillium rubens*, and *Penicillium* sp. were retrieved from all three flight samples treated with PMA. Similarly, metagenome sequences corresponded to eight bacterial species (*Enterobacter bugandensis*, *Klebsiella pneumonia*, *Pantoea conspicua*, *Pantoea dispersa*, *Pantoea* sp., *Staphylococcus aureus*, *Staphylococcus epidermidis*, and *Staphylococcus saprophyticus*) were found in all the three flights. Three species *Arthrobacter russicus*, *Arthrobacter siccitoleran*, and *Micrococcus yunnanensis* that were isolated via cultivation were not seen in PMA-treated and untreated samples. However, in total, sequences from 403 microbial species (Additional file 2: Table S2) were retrieved, constituting > 90% as uncultivable with the cultural conditions employed.

### Functional analysis

**Fig. 4:**
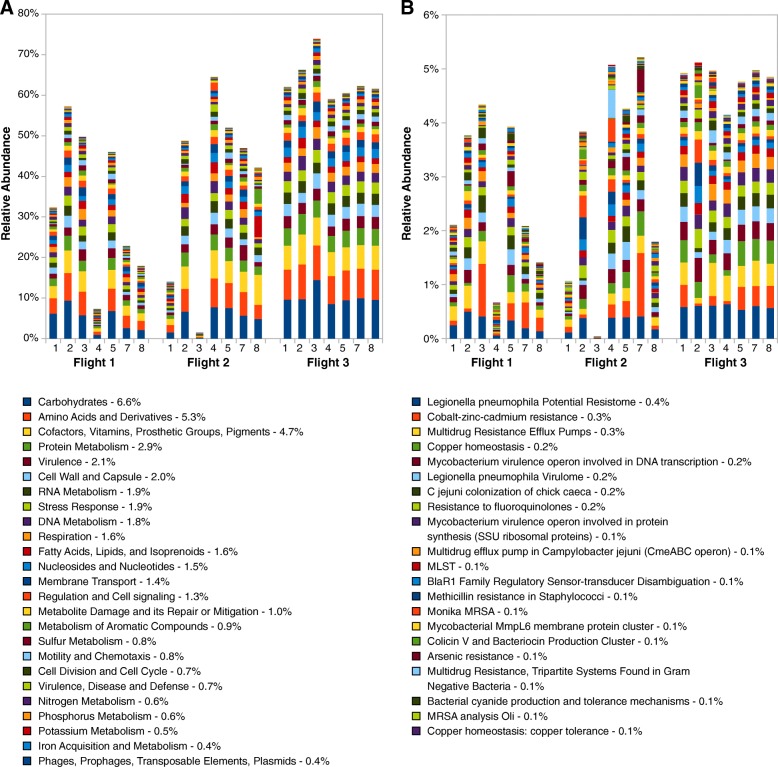
Relative abundance of metagenomics reads associated with **a** metabolism and **b** virulence

Reads associated with carbohydrate metabolism, amino acid derivatives and cofactors, vitamins, etc. were the highest among all three flights (Fig. 4a) (Additional file 6). The relative abundance of reads associated with various metabolic functions was similar between Flights 1 and 3 (ANOSIM, *R* = 0.4, *p* = 0.05) and between Flight 2 and Flight 3 (ANOSIM, *R* = 0.3, *p* = 0.05). When comparing Flights 1 and 2, the functional pathways were indistinguishable from one another (ANOSIM R = 0). The Mann-Whitney-Wilcoxon statistical analyses found no significant difference between any of the flights (*p* = 0.05).

### Virulence profile

Metagenomic reads from all flights were grouped for different virulence categories (Fig. 4b) using the reference virulence factors in the SEED database. Computational analyses showed that the *Legionella* resistome, cobalt-zinc-cadmium resistance, and multi-drug-resistant (MDR) resistance efflux pump were high on all flights and all locations. A uniform trend for virulence factors was seen for all flight samples. Virulence factors in samples were similar when comparing Flights 1 and 3 and Flights 2 and 3 (*R* = 0.3 and *R* = 0.2, respectively, *p* ≤ 0.05). Virulence factors observed from Flight 1 and Flight 2 did not show this pattern and appeared indistinguishable from each other as that of functional pathway profiles (*R* = 0). The Mann-Whitney-Wilcoxon statistical method did not detect a significant difference in virulence factors sampled among all three flights (*p* > 0.05).

### Antimicrobial resistance profile

**Fig. 5:**
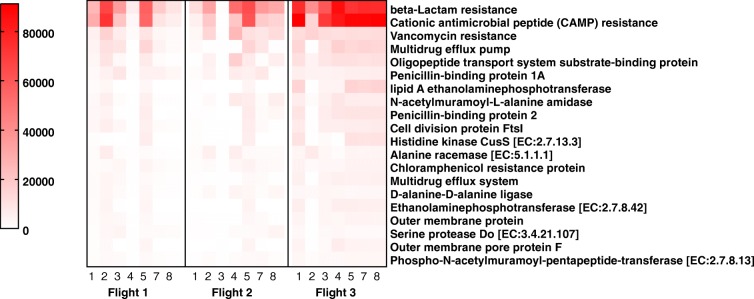
Distribution of antibiotic resistance across samples as seen in metagenomics analysis. Read counts of AMR genes grouped together based on the class of antibiotic they are resistant to, as detected by metagenomics. White boxes indicate genes that were absent in a particular sample. Red indicates the highest read count and pink, the lowest read count. Columns represent samples and rows represent antibiotic resistance features

AMR signatures were organized into four major categories: (i) beta-lactam resistance, (ii) cationic antimicrobial peptide (CAMP) resistance, (iii) vancomycin resistance, and (iv) other antimicrobial resistance genes such as MDR efflux pump, penicillin-binding proteins, and chloramphenicol resistance (Fig. 5). Total reads associated with AMR in Flight 3 increased by twofold when compared with Flights 1 and 2 (Additional file 3: Figure S6; Additional file 4: Table S4). The AMR resistance factors overlapped with several other virulence factors (e.g., MDR efflux pump). However, as the virulence genes were independent and did not overlap, both analyses (AMR and virulence) were independently performed and presented. Comparatively, fewer reads (~ 50%) were classified into AMR than the virulence category, (Additional file 3: Figure S7). Collective beta-lactam resistance derived from the metagenome shows that physical (*OmpF*, *OmpC*), transformational (penicillin-binding protein), and degradational (*AmpC*), and MDR efflux pump (OMP, RND, MPF) mechanisms were allocated by the microorganisms on the ISS.

### Prevalence of BSL-2 pathogens

**Fig. 6:**
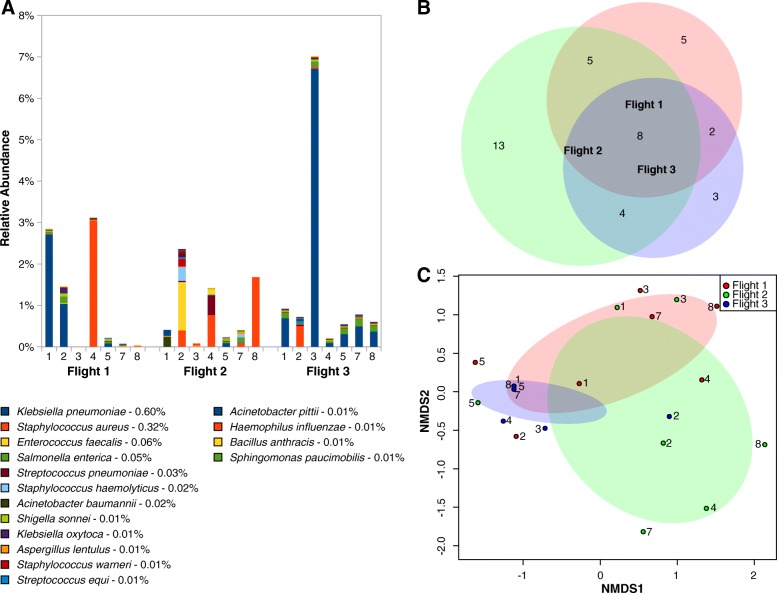
**a** Abundance profile of biosafety level 2 bacterial and fungal organisms in the ISS microbiome based on metagenomics sequences of the PMA-treated samples. **b** Venn diagram representing the common BSL-2 species between all three flights. **c** Species-based NMDS analysis representing various sampling sites in 2D ordinate as per the microbiome composition

**Fig. 7:**
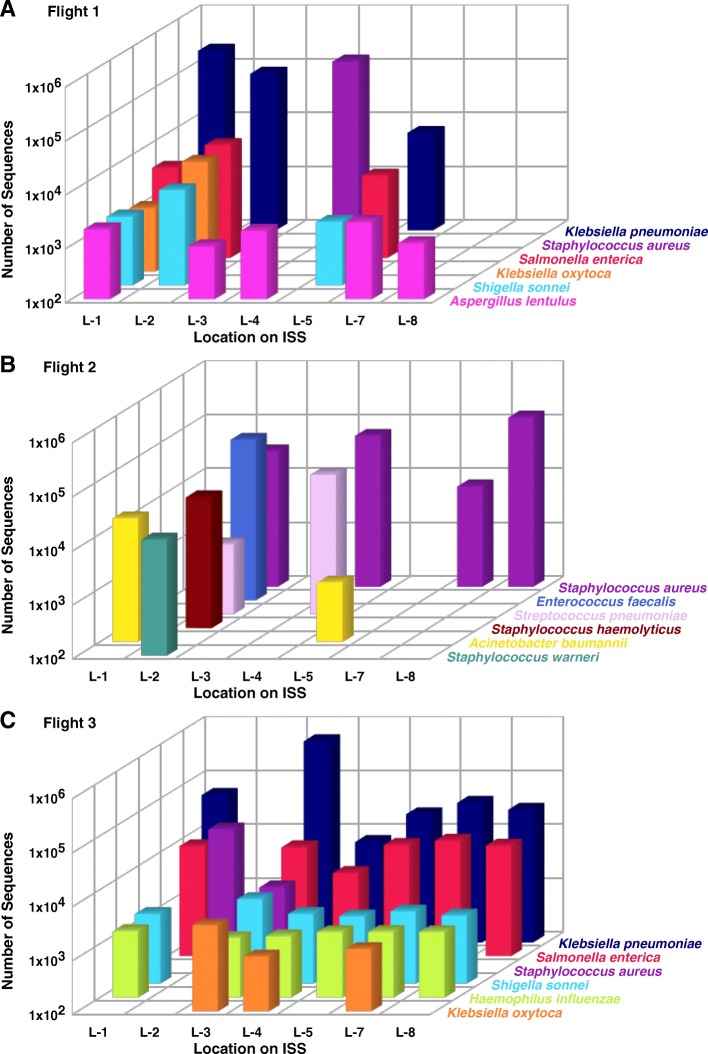
Dominant and persistent BSL-2 microbial species of ISS environmental surfaces on **a** Flight 1, **b** Flight 2, and **c** Flight 3

To further access the risk to human inhabitants, BSL-2-associated bacterial and fungal metagenomics sequences of the PMA-treated samples were mined (Fig. 6 a-c). *Klebsiella pneumoniae*, *Staphylococcus aureus*, *Enterococcus faecalis*, and *Salmonella enterica* were the dominant BSL-2 organisms identified from PMA-treated samples. Among the BSL-2 microbes observed, eight species were found in all three flights, they were as follows: *Acinetobacter baumannii*, *Haemophilus influenza*, *K. pneumonia*, *Salmonella enterica*, *Shigella sonnei*, *Staphylococcus aureus*, *Yersinia frederiksenii*, and *Aspergillus lentulus* (Fig. 6b). When compared with the species description of the ISS microbiome, *K. pneumoniae* and *S. enterica* tended to co-occur and were the dominant species at most sampling locations of Flight 3 (Fig. 7c). Significant similarity was detected among BSL-2 pathogens detected within Flight 2 and Flight 3 (ANOSIM *R* = 0.3, *p <* 0.05), as well as within Flight 1 and Flight 3 (ANOSIM *R* = 0.2, *p* = 0.059). BSL-2 microbes in common include *K. pneumoniae*, *S. aureus*, and *S. enterica.* However, no similarity was detected between Flight 1 and Flight 2 (ANOSIM *R* = 0). The Mann-Whitney-Wilcoxon statistical analyses detected significant differences between BSL-2 pathogens within Flight 2 and Flight 3 (*p* < 0.05) as well as within Flight 1 and Flight 2 (*P* < 0.05). Even though ANOSIM detected similarity, the Mann-Whitney-Wilcoxon test showed no difference between Flight 1 and Flight 3 (*p* ≥ 0.05). NMDS analysis showed locations that share a similar set of BSL-2 organisms tend to aggregate together in the ordination space, especially in the Flight 3 sampling locations (Fig. 6c).

### Succession and persistence of key microbial communities

Dominant and persistent microbial genera of ISS environmental surfaces sampled from all three flights treated with PMA are given in Additional file 3: Figure S5. *Pantoea* species were found to overtake the ISS microbiome and became the dominant genus in samples from Flight 3. Other genera that showed noticeable persistence at the flight level were *Klebsiella*, *Staphylococcus*, *Erwinia*, and *Penicillium.* The dominance of *Pantoea* was clearly documented in this study for all three flights at location #5 (Surface rack). PMA-treated samples from locations #1 and #4 showed a pattern where *Penicillium* species were dominant and persistent in Flight 1 and Flight 2, but later on, reads of *Pantoea* species were found in higher proportions in Flight 3. The same pattern could not be observed in the samples collected from location #3 (ARED platform) in the first two flights, whereas *Klebsiella* dominated in Flight 3 samples. The reads of *Methylobacterium* were overwhelmingly present in Flight 1 and Flight 2 within location #7 (LAB103), but in Flight 3, reads of *Pantoea* became more abundant. The wall of the crew quarters (location #8) showed the presence of spore-forming fungi (*Penicillium*) in Flight 1 and bacteria (*Paenibacillus*) in Flight 2, but again ,*Pantoea* species were retrieved in high numbers in Flight 3. Unlike other locations, location #2 (space toilet) did not show a similar change in its microbial composition over the three flight sampling events. However, *Staphylococcus* species were shown to be present in high numbers during Flight 2 and Flight 3 in PMA-treated samples collected from location #2. Irrespective of locations sampled, *Pantoea* species were found to be dominant in five out of seven Flight 3 locations sampled.

The BSL-2 microbial species that were persistent and exhibited succession phenomenon of various ISS environmental surfaces of all three flight samples are depicted in Fig. 7. The incidence of the top 10 BSL-2 pathogens and the number of reads retrieved are depicted in Table 2. The incidence of BSL-2 pathogens varied in numbers, with a notable persistence of members of *Enterobacteriaceae* in Flight 1 (> 50% of BSL-2 taxa) and Flight 3 (> 90% of BSL-2 taxa). However, *Staphylococcus* species were the most dominant BSL-2 taxa detected in Flight 2 (> 50%). *Klebsiella pneumoniae* reads were the most abundant in Flight 1 (~ 50% of the top 10 BSL-2 taxa reads) and Flight 3 (> 80% of the top 10 BSL-2 taxa reads), but this nosocomial opportunistic pathogen dropped to the 11th position in Flight 2 (6856 reads).

**Table 2:** The abundance of metagenomics sequences associated with top 10 risk group or BSL-2 microbial pathogens of ISS environment

BSL-2 pathogen	Flight 1	BSL-2 pathogen	Flight 2	BSL-2 pathogen	Flight 3
*Klebsiella pneumoniae*	305,176	*Staphylococcus aureus*	236,354	*Klebsiella pneumoniae*	689,778
*Staphylococcus aureus*	246,965	*Enterococcus faecalis*	94,078	*Salmonella enterica*	61,562
*Salmonella enterica*	20,669	*Streptococcus pneumoniae*	40,284	*Staphylococcus aureus*	43,724
*Klebsiella oxytoca*	12,572	*Staphylococcus haemolyticus*	26,486	*Shigella sonnei*	13,530
*Shigella sonnei*	9407	*Acinetobacter baumannii*	20,781	*Haemophilus influenzae*	9289
*Aspergillus lentulus*	8748	*Staphylococcus warneri*	14,323	*Klebsiella oxytoca*	6456
*Yersinia frederiksenii*	2231	*Acinetobacter pittii*	11,496	*Streptococcus equi*	5615
*Trichosporon asahii*	2170	*Bacillus anthracis*	10,151	*Yersinia enterocolitica*	5072
*Staphylococcus hominis*	1905	*Sphingomonas paucimobilis*	10,119	*Raoultella ornithinolytica*	4114
*Kosakonia radicincitans*	1571	*Enterococcus faecium*	7196	*Acinetobacter baumannii*	3753

In total, 17 bacterial species were persistent in location #5 (Node 1) and were successively present in all three flights (Table 3). The reads of *K. pneumoniae* were consistently higher than other BSL-2 taxa and continued to be retrieved in all three flight sampling periods. Other noticeable opportunistic pathogens in location #5 of all flights were *Acinetobacter baumannii*, *Enterobacter cloacae*, *Salmonella enterica*, and *Shigella sonnei*. The reads of *Staphylococcus saprophyticus*, a non-pathogenic skin bacterium, were consistently retrieved from location #7 (LAB) during all three flights (Additional file 2: Table S2). Among fungal reads, six fungi showed persistence in all three flights and were not restricted to location #5 as documented for bacterial persistence. Notably, the reads of *Penicillium rubens*, a saprophytic fungus, were present in high numbers throughout all three sampling events, but also from locations #2, #3, and #8. *Rhodotorula* sp. JG-1b, a benign fungus, was sequenced in high numbers at location #2 (space toilet). None of the pathogenic fungi was persistent in any of the locations sampled.

**Table 3:** Location-wise persistent microbial taxa of the ISS environmental surfaces

Microbial taxa	Domain	Flight 1	Flight 2	Flight 3	Location	BSL level
*Acinetobacter baumannii*	Bacteria	1340	1279	1039	5	2
*Enterobacter cloacae*	Bacteria	4774	4937	5876	5	2
*Escherichia coli*	Bacteria	11,300	10,766	17,905	5	1
*Klebsiella pneumoniae*	Bacteria	6476	6856	23,873	5	2
*Pantoea agglomerans*	Bacteria	28,842	30,016	10,467	5	1
*Pantoea ananatis*	Bacteria	34,829	39,399	41,268	5	1
*Pantoea conspicua*	Bacteria	571,313	568,170	6544	5	1
*Pantoea dispersa*	Bacteria	1053	1220	3718	5	1
*Pantoea* sp. 3.5.1	Bacteria	48,025	51,212	927	5	1
*Pantoea* sp. AS-PWVM4	Bacteria	944	1031	1973	5	1
*Pantoea* sp. At-9b	Bacteria	1027	1072	4879	5	1
*Pantoea* sp. OV426	Bacteria	6174	6797	1475	5	1
*Pantoea* sp. OXWO6B1	Bacteria	1336	1400	13,885	5	1
*Pantoea stewartii*	Bacteria	1402	1388	3807	5	1
*Pantoea vagans*	Bacteria	8073	8819	48,922	5	1
*Salmonella enterica*	Bacteria	3399	3634	11,539	5	2
*Shigella sonnei*	Bacteria	1537	1738	1793	5	2
*Staphylococcus saprophyticus*	Bacteria	1181	60,572	1487	7	1
*Aspergillus niger*	Fungi	62,208	1428	7522	1	1
*Penicillium camemberti*	Fungi	5609	19,577	3341	3	1
*Penicillium chrysogenum*	Fungi	22,685	65,566	2119	3	1
*Penicillium nordicum*	Fungi	6330	20,672	878	3	1
*Penicillium rubens*	Fungi	4341	384,342	212,282	2	1
*Penicillium rubens*	Fungi	185,166	529,688	17,693	3	1
*Penicillium rubens*	Fungi	192,495	11,834	1513	8	1
*Rhodotorula* sp. JG-1b	Fungi	7613	77,458	400,490	2	1

The number of *K. pneumoniae* reads collected from the location #5 samplings of Flight 1 and Flight 2 (~ 6.5 × 10^3^ reads) was lower than those collected from the Flight 3 samples (2.4 × 10^4^ reads). *Aspergillus lentulus* and *K. pneumoniae* dominated in Flight 1 samples (Fig. 7a), *S. aureus* dominated in Flight 2 samples (Fig. 7b), and *K. pneumoniae* dominated in Flight 3 samples collected from locations #7 and #8 (Fig. 7b). However, the other locations (#1, #2, #3, and #4) did not have any patterns with respect to the abundance of BSL-2 microorganisms. Irrespective of locations sampled, *K. pneumoniae* species were found to be dominant in six out of seven locations sampled in Flight 3 (Fig. 7c). A high abundance of *K. pneumoniae* sequences in Flight 1 at locations #1 and #2 (Fig. 7a), as well as the absence of these reads in Flight 2 except at location #5 (Fig. 7b), was noticed.

As previously mentioned, *Pantoea* sequences were retrieved in higher abundance, successively becoming dominant in the Flight 3 samples, and hence, a comparative study was carried out to find all the virulence factors identified in this species. The whole genome sequence of a cultivated *Pantoea* IF5SW-P1 strain [19] was mined and compared with the metagenomics virulence analysis of Flight 3 samples. The resulting analyses identified 66 out of 85 virulence factors of the *Pantoea* IF5SW-P1 strain from metagenome sequences of Flight 3 (Additional file 5: Table S3).

### Comparative metagenome sequence analyses and core microbiome of various ISS components

**Fig. 8:**
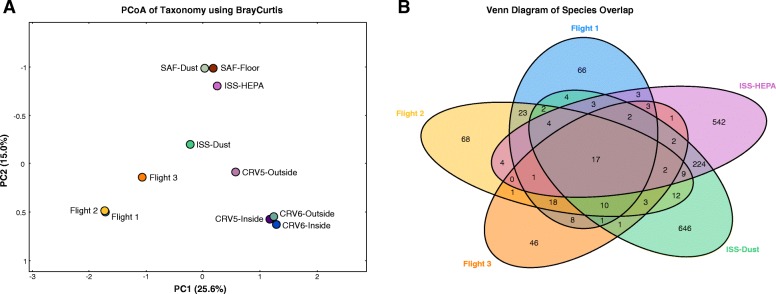
**a** PCoA plot depicting dissimilarity between the ISS samples and associated microbiome. **b** Venn plot visualization of common species found in Flight 1, Flight 2, Flight 3, ISS dust, and ISS-HEPA

Comparative metagenome sequence analyses showed that the intact microbiome of the ISS environmental surfaces (Flight 1, Flight 2, and Flight 3) tend to align together but separate from other samples, including ISS dust and ISS-HEPA, suggesting that their microbial composition is distinct from one another (Fig. 8a). Dominant species were different for all ISS components (surface, *Pantoea*; dust, *Staphylococcus*; HEPA, *Corynebacterium*), SAF (*Acinetobacter*), and Commercial resupply vehicle-CRV (*Bacillus*) sample sets representing diverse environmental conditions on each surface. The present study attempts to describe the ISS core microbiome based on the total metagenome analysis reported to date, which has been visualized as a Venn diagram (Fig. 8b) where each ellipse represents a sample set and the core is represented by the overlapping microbiome of each ellipse. The Venn diagram (Fig. 8b) represents an analysis of microbial species unique to a particular habitat (Flight 1, 66 species; Flight 2, 68 species; Flight 3, 46 species; ISS-HEPA, 542 species; and ISS dust, 646 species) but also the shared presence forming the core microbiome (17 species). The ISS core microbiome was dominated by fungi (*Penicillium brasilianum*, *P. chrysogenum*, *P. digitatum*, *P. expansum*, *P. freii*, *P. griseofulvum*, *P. roqueforti*, *P. rubens*, *Aspergillus calidoustus*, and *A. niger*) and bacteria (*Cutibacterium acnes*, *Enterobacter cloacae*, *Escherichia coli*, *Pantoea ananatis*, *Salmonella enterica*, *Staphylococcus aureus*, and *S. epidermidis*) shared by all the samples but at different proportions.

## Discussion

Shotgun metagenome analyses of samples collected over time provides not only the taxonomic profile, but also an in-depth understanding of microbial dynamics at the species or strain levels and a functional profile of a given sample. Studies of ISS metagenomes will help NASA in setting long-term strategies for space travel and facilitate the development of microbial contamination reduction regimes through periodic maintenance. Functional analyses will assist in risk assessment and countermeasure designs. One of the basic advantages of shotgun metagenome analysis is its efficiency in detecting non-dominant populations present in an active gene pool.

Humans have always contributed to the built environment by dispersal of human-associated microorganisms (e.g., through the shedding of skin cells, sneezing, coughing). Since the ISS is a closed system, the environment external to the ISS does not contribute to the microbial load; hence, human activities inside the closed system and cargo shipped were the major contributors to the microbial population. Relatively low numbers of reads from human origin (~ 4%) were observed in non-PMA-treated samples, whereas ~ 96% of the metagenomics reads were associated with microorganisms. In addition, the number of human reads in non-PMA-treated samples were higher (~ 1.7 × 10^6^ reads) compared to the PMA-treated (~ 77 × 10^3^ reads) samples. The effectiveness of PMA treatment in removing genetic materials associated with dead cells or compromised cell membranes has been demonstrated here and elsewhere [31, 34, 35].

Multiple studies have reported on the microbial composition of built environments using gene-targeted amplicon sequencing of bacteria and fungi populations. The ISS is a hermetically sealed closed system with no volumetric exchange of air with the external; hence, comparisons with relatively open systems like offices [56], homes [57], and hospitals [58] may not provide the ideal candidates for comparison, and hence were not included in this study. The microbiomes (amplicon sequences resolvable to family level) of ISS comparable airtight closed systems such as lunar/Mars analogous habitat (ILMAH) exhibited the high abundance of *Staphylococcaceae*, *Corynebacteriaceae*, *Caulobacteraceae*, *Pleosporaceae*, and *Sporidiobolaceae* [59, 60]. A similar closed system, Mars 500, that analyzed only bacterial composition showed a high abundance of sequences assigned to *Corynebacteriaceae*, *Burkholderiaceae*, and *Staphylococcaceae* [61]. However, the present metagenomics study revealed a high abundance of sequences from *Pantoea* (*Enterobacteriaceae*), *Methylobacterium* (*Methylobacteriaceae*), *Staphylococcus* (*Staphylococcaceae*), *Penicillium* (*Aspergillaceae*), and *Rhodotorula* (*Sporidiobolaceae*). Based on these microbial compositions, it is evident that the ISS environmental surfaces were not similar to Earth-based analogs except for the presence of human skin-associated members of the family *Staphylococcaceae* and environmental yeast, *Sporidiobolaceae*. Since ILMAH and Mars 500 habitat studies did not produce shotgun metagenome data, they were not included in the comparative analyses. The present shotgun metagenome study was an improvement on previous amplicon-targeted microbiome studies reported about the ISS or its analogs [32, 62]. Metagenome data generated in this study provided more sequence coverage than the amplicon-targeted ISS microbiome studies [62], providing a higher resolution of the microbiome composition and the functional makeup of the ecosystem.

The comparative metagenome sequence analyses revealed separate grouping for the ISS environmental surfaces (Flight 1, Flight 2, and Flight 3) from other samples (Fig. 8a). This might be due to the sample collection period of the ISS (1 day collection for ISS dust vs 40 months collection of ISS-HEPA) where microorganisms could have been introduced at various time points. The ISS microbiome of the environmental surfaces was different among themselves, yet Flight 1 and Flight 2 showed compositional similarity, whereas Flight 3 converged towards a system dominated by *Pantoea* species. Compositional variation due to the convergence to *Pantoea* species (Fig. 7) and a lowering of species richness placed Flight 3 (Fig. 2; Shannon/Chao indices) away from Flight 1 and Flight 2, but still in near ordinate positioning. This represents a classic example of microbial succession where the domination of one species leads to the reduction in species richness and a shift in ecosystem microbial composition that was evident in Flight 3.

In contrast to the ISS microbiome (surface, *Pantoea*; dust, *Staphylococcus*; HEPA, *Corynebacterium*), Earth-originated CRV (*Bacillus*) and SAF (*Acinetobacter*) sample sets were placed in a different ordinate position showing the compositional dissimilarity between the two. Even though CRV (Florida) and SAF (California) were from NASA cleanroom facilities, unique microbial compositions were observed, which might be attributed to the different geographical locations as reported earlier [63]. The variation in microbial composition, succession, and persistence could be the result of the stressors acting on them. These stressors might be associated with physical characteristics of the sample sets, but are not limited to microgravity (ISS surface and ISS dust), desiccation (ISS-HEPA, SAF), and oligotrophic conditions (SAF, CRV). The difference in the ISS surface microbiome compared to the other sampled microbiomes confirmed that the influence of forwarding contamination to ISS via CRV or processing cleanrooms such as SAF was minimal.

Unlike other ISS surface samples, the microbiome of intact cells of the ISS-HEPA habitat exhibited similarities with the microbiomes of the SAF dust or SAF floor surfaces. The ISS-HEPA was 40 months old, desiccated, and a low nutrient bioavailability habitat, which could be similar to stressed conditions of the SAF environment. Even though dominant microbes were different in these systems, the core microbiomes were similar. An interesting observation was the distinctive position of ISS dust in the ordinate space, where the ISS dust was collected from a vacuum bag consisting of particles vacuumed for only 1 day. These samples contained human skin-associated microbes dominated by *Staphylococcus* species and food spoilage fungi such as *Penicillium* species. The ISS dust sample possessed not only particulates but also food remnants and their genes (*Zea mays*, *Oryza sativa*, *Pisum sativum*, *Arachis hypogaea*, etc.) which might have selectively allowed the proliferation of *Staphylococcus* and *Penicillium* species.

Since PCoA plots confirmed that SAF and CRV microbiomes were different from ISS (Fig. 8a), only shotgun metagenomes of the ISS were compared to elucidate the core ISS microbiome. In general, understanding the community dynamics of the core microbiome (persistence) or common members of different components of a closed system might help to elucidate their influence over the deterioration of the habitat or their effect on the health of the inhabitants. Despite different sampling times, methods, and processing techniques, certain microbes were unchanging in the ISS microbiome. The core microbiome is the stability factor and is responsible for withstanding the entropy of an active system like the ISS. A long-term study of the ISS core microbiome should be undertaken to understand the founding structure of the ISS microbial ecology, which will help NASA to regulate beneficial microbes and restrain problematic microbes when needed. More research is warranted to develop countermeasure solutions to selectively eradicate problematic microbes without disturbing beneficial microbes, e.g., with phage treatment [64]. Microbes are continuously adapting to changing habitat and niches. Since humans and cargos were constantly moved in and out of the ISS, knowledge of the ISS core microbiome will help NASA to maintain its biological integrity in line with its structural integrity.

The ISS microbiome represents a “minimal core” model hypothesized based on the large sets of human microbiome data [65], in which all the human subjects shared few microbial species, large overlaps were found in subsets but a very little was common between all the sets. This was not the case for the built indoor microbiome studies based on Earth, where it was reported that the normal range of indoor environmental conditions might not be large enough to impact microbial communities [56].

The study of rare microbes helps us to understand the functional diversity of a community, which would have been missed in an amplicon-based study [66]. One of the observations in the functional analysis of the ISS is its similarity across the flights and samples (Fig. 4a) which is indicative of a stable core. In this type of system, microbes can take the role of other microbes performing similar functions, which is a good example of the “insurance hypothesis” [67]. In the insurance hypothesis model, it is assumed that more than one organism performing a similar function can act as a buffer against system entropy. Under the unfavorable conditions of microgravity, when one microorganism is eliminated, the other microbes adapted to the microgravity condition can replace it to perform the required functions (e.g., adherence). Such adaptations will also help in the persistence of microbes since a system devoid of persistent microbes will fail to have a stable core microbiome. Even though interference of microbes associated with cargos and humans cannot be ruled out, a stable healthy microbial ecosystem is an essential surviving principal for established microbes. In metagenome analysis of ISS environmental surfaces (not dust or HEPA), *Pantoea* and *Klebsiella* species showed not only persistence but also exhibited succession across samples collected from three flights (~ 1.5 years) and hence should be considered as true persistors [68].

### Dominant microbiome of various ISS components

The ISS air is circulated into the cabin after a revitalization process, and hence, the microbiome of air particulates of the HEPA system might influence the microbiome of ISS environmental surfaces. Unlike the present study, *Corynebacterium* species were dominant in the ISS dust and HEPA [35], but in both cases, *Staphylococcus* members were found to be present in high numbers. This might be due to the fact that the ISS-HEPA system could eradicate the members of *Pantoea* and *Methylobacterium*, which were reported to be susceptible to desiccation [69], whereas *Staphylococcus* species might withstand the low moisture condition.

Retrieval of *Rhodotorula* sequences in high numbers (35% relative abundance) and its similarity with the whole genome sequences of the psychrotolerant *Rhodotorula* sp. JG1b strain isolated from the permafrost in the hyper-arid McMurdo Dry Valleys of Antarctica [70] warrant more study. A high-quality genome of this strain was also retrieved from the metagenome and its annotation is underway. Furthermore, 33 strains of *Rhodotorula* sp. were cultured from these ISS samples [55], and whole genome sequencing of these strains is necessary when comparisons are made. *Rhodotorula* sp. JG1b sequences were retrieved consistently from location #2 in all three flights (Table 2).

### Persistence of BSL-2 pathogens

Across three flight samplings, *K. pneumoniae* reads, an opportunistic BSL-2 pathogen [71], were retrieved from locations #1, #2, and #5 during Flight 1, and successively, its reads persisted in location #5 of Flight 1 and 2. Subsequently, in Flight 3, except at location #2, all other locations showed the presence of this opportunistic pathogen. To determine whether all these reads came from the same *K. pneumoniae* strain, more detailed analyses are needed such as source tracking [72] and extracting the genome from the metagenome reads [73]. The preliminary analyses confirmed that the 5.3 Mb draft genomes of *K. pneumoniae* retrieved from the Flight 1, location #1 (2.17 × 10^5^ shotgun reads), and Flight 3, location #3 (5.36 × 10^5^ reads), were identical (99% average nucleotide index), but the in-depth characterization is warranted. The absence of *K. pneumoniae* reads in Flight 2 locations except location #5 might be due to the cleaning regime followed by the crew. Alternatively, the stowed experimental materials at location #5 were not in use between Flights 1 and 2. *K. pneumoniae* observed at location #5 of Flight 1 could be the etiological agent, which might have spread across the other locations sampled during Flight 3. One of the explanations could be location #5, being a stowage unit, might not have been disturbed as frequently as other locations sampled where day-to-day activities were high due to various planned experiments or informal social gathering (location #1, Cupola), exercising (location #3, ARED platform), food (location #4; dining table), etc. During the 1-year span between Flight 2 and Flight 3, the stowed materials from location #5 might have been moved to other places of the ISS due to the implementation of various experiments, which could have led to the spread of the *K. pneumoniae*. It has been reported that the accumulation and persistence of microbial populations might be affected by the nature of the materials used to construct this closed habitat [74]. Detailed logs of various experimental procedures should be carefully looked into before coming to this conclusion, and at present, such data are not made available for this study. Another probable reason could be that the cargo might be the contamination vector rather than the crew as no *K. pneumoniae* reads were retrieved from location #2 (space toilet) of Flight 3 and all sampled locations of Flight 2 except location #5. Maximum crew activities were noticed at locations #1, #2, #3, #4, and #8 while the other locations, #5, #6, and #7, were not used as frequently. It is predicted that a study of these locations would give a holistic microbial profile of ISS and its influence on humans. However, the statistical analyses showed no correlation among these sets of locations.

In addition to *K. pneumoniae*, the genetic signature of *Pantoea* was found in all three flight samples, which supports its biological persistence on the ISS environmental surfaces. Since members of *Pantoea* were reported as an opportunistic pathogen of both humans and plants [75], their presence in higher numbers and persistence might hinder long-duration human stay in a closed system because both in situ food production and human health could be impacted. Isolation of *Pantoea* species [55] and the whole genome sequences of ISS strains were reported [19], and in-depth analyses of genomes extracted from *Pantoea* reads are underway.

### Functional properties of the ISS microbiome

This is the first report of microbial succession reported at the ISS with an in-depth analysis of AMR and virulence profiles. In treating bacterial infectious diseases, β-lactam antibiotics were widely used, which subsequently lead to the development of resistance in target organisms [76]. The β-lactamase that inactivates carbapenems and β-lactamase inhibitors were reported to be prevalent around the world, and resistance to the new antibiotics, which were designed to overcome β-lactam resistance, had already emerged within a year [77]. It has been extensively reviewed that penicillin-binding proteins (PBP), membrane-spanning porin proteins (*OmpF* and *OmpC*), would bind with some β-lactam antibiotics and physically alter them [78], and some other classes of β-lactams when passed through PBP interactions could get removed by efflux pumps [79]. Furthermore, the *AmpC* gene was reported to hydrolyze certain compounds, like penicillin and cephalosporin, but could not degrade all kinds of antibiotics [80]. Hence, bacterial pathogens could alter the β-lactam targets in multiple ways [76] and could acquire resistance to several antibiotics [81]. In this study, AMR gene categories pertaining to the outer membrane proteins (*OmpF* and *OmpC*; two of the most common porins), transformation proteins (PBP), degradation (*AmpC*), an efflux pump (OMP, RND, MPF) were retrieved in high numbers.

Approximately one million reads of β-lactamase-resistant AMR genes were retrieved and prevalent in Flight 3 samples. As reported in this study, the AMR gene categories uniquely identified in ISS dust samples [35] were assigned to genes related to the ATP-binding cassette superfamily, multidrug and toxic compound extrusion family, rRNA methyltransferase, methionine sulfoxide reductase (*msr*A), fluoroquinolone resistance (*pat*A and *pat*B), and clindamycin resistance (*erm*ABC). Sequences of *K. pneumoniae* found in high numbers in location #5 in all three flights and its lateral spread throughout the locations during Flight 3 needs to be studied in detail. However, the mere presence of AMR genes from the intact cells in PMA-treated samples would not endorse the involvement of their pathogenic potential but expression analyses utilizing proteomics/transcriptomics are required to confirm the pathogenesis. The isolation and archival of several *Enterobacteriaceae* members, including MDR *K. pneumoniae* strains, in a parallel study from the same samples [55] and future research characterizing molecular mechanism(s) would shed limelight into the microbial pathogenicity of these ISS isolates.

Antibiotic-resistant proteins associated with *K. pneumoniae* (e.g., carbapenemase, known as KPC complex) were reported to possess a broad substrate profile, including penicillins, cephalosporins, carbapenems, and β-lactamase inhibitors [82]. The amino acid substitutions in KPC-2 revealed increased susceptibility to β-lactamase inhibitors and β-lactams, indicating that the *K. pneumoniae* β-lactamase complex was responsible for hydrolyzing a wide variety of antimicrobials [83]. In two relevant studies where isolation of *K. pneumoniae* strains from these ISS locations [55] and its phenotypic resistances against multiple drugs (cefazolin, cefoxitin, ciprofloxacin, erythromycin, gentamicin, oxacillin, penicillin, rifampin, and tobramycin) (Checinska Sielaff et al. 2018; submitted) warranted development of suitable countermeasures in eradicating the etiological agents. Moreover, additional sampling events in subsequent years from these locations will also reveal whether existing ISS maintenance using systematic cleaning regimes would be sufficient to remove the persistent microorganisms.

It is also evident from previous studies that microbes tend to increase virulence in microgravity [84]. In this study, we observed a continuous increase in the number of metagenomics reads associated with virulence from Flight 1 to the maximum in Flight 3 in PMA-treated samples (Additional file 3: Figure S7). Major virulence factors identified were *Legionella pneumophila* potential resistome (6 × 10^5^ reads), cobalt-zinc-cadmium resistance (4.8 × 10^5^ reads), copper homeostasis (3.4 × 10^5^), and Mycobacterium virulence operon (3.3 × 10^5^). Even though there were no reads associated with *Legionella pneumophila* in species identification, its resistome [85–87] is very similar to other gram-negative bacteria like *Pantoea* [88] and *Klebsiella* [89] and is well defined in SEED database, which could be the plausible reason for the identification of the reads. Divalent cations such as Co^2+^, Zn^2+^, and Ni^2+^ are essential for bacteria but are toxic in higher concentrations [90]. These redox-active metals, due to unfilled d-orbitals, cycle between oxidation states, supporting the metal homeostasis system. This is crucial in deciding bacterial host interface, by determining the activity of metal-responsive transcriptional regulatory networks in microbial pathogens [91, 92]. This mechanism helps bacteria to adapt to the host metal homeostasis variation, a defense mechanism against bacteria in case of infection. Bacteria evolved to overcome this host defense mechanism by developing various metal resistance mechanisms [93]. In light of recent studies where the human gene expression related to the immune system, DNA repair, bone formation networks, hypoxia, and hypercapnia changed considerably in microgravity (unpublished), and also the incidence of various infections on the ISS [10], could be the plausible reason for high read incidence for cobalt-zinc-cadmium resistance. Major genes identified for the metal resistance were cadmium-transporting ATPase (EC 3.6.3.3); cation efflux system protein *CusC* and *CusF* precursors; cobalt-zinc-cadmium resistance protein *CzcA*, *CzcB* and *CzcD*; copper sensory histidine kinase *CusS*; heavy metal-resistant transcriptional regulator *HmrR*; heavy metal RND efflux *CzcC*, and zinc transporter *ZitB*, to name a few. Multidrug-resistance efflux pump was a crossover from AMR analysis contributing to virulence. Another known human pathogen, *Mycobacterium*, has been known to colonize hosts without any adverse effect, especially the non-tuberculosis *Mycobacterium* (NTM) [94]. Even though species identification showed very few hits for *Mycobacterium abscessus*, which is an NTM, the functional analysis showed higher numbers of reads for *Mycobacterium* virulence operon involved in DNA transcription. Most of the BLAST hits under this category were identified as a DNA-directed RNA polymerase beta subunit (EC 2.7.7.6), which has undergone specific mutation rendering them resistant to antibiotics [95] via a common variation (Gln ➔ His) at codon 513 [96]. The SEED database does not specifically identify the mutation but provides collective information about the various *Mycobacterium* virulence operons. Other *Mycobacterium* virulence operons identified from Flight 1 to Flight 3 were involved in fatty acids biosynthesis, lipid degradation, protein synthesis, and dormancy regulation. Further analysis is required to study how *Mycobacterium* is evolving and interacting with their hosts in microgravity. A complete list of virulence factors is provided in Additional file 4: Table S4.

Although further experiments are required to conclude a correlation between microbial space adaptation and increase in virulence, this study gives a deeper insight of the ISS environment and provides a notion that future sampling should include human samples. By including human samples, a complete picture at the functional level could be generated about how the ISS environmental microbiome is affected by humans and vice versa. To understand the role of microgravity on the functional profile (AMR and pathogenesis) of microbial communities, more research is warranted since sophisticated molecular methods are required to measure biological entities (single cell genomes, genome extraction from metagenomes) with appropriate ground controls, unified metadata generation, and computational power to analyze large datasets.

## Additional files

Additional file 1: ** Table S1.** Initial DNA concentration and library yield for metagenomics analyses. (DOCX 16 kb)

Additional file 2: **Table S2.** Microbial species identified based on metagenomic reads in all flights and locations. (XLSX 76 kb)

Additional file 3: **Figure S1.** Percentage of total reads obtained from PMA and non-PMA treated samples. **Figure S2.** Proportional abundance at flight level. The proportional abundance of different flight sampling shows the presence of all domains (Bacteria, Archaea, and Eukaryota). There is a noticeable increase in bacterial population from Flight 1 to Flight 3. **Figure S3.** Top 25 species dominating the microbial composition of ISS samples. Normalized sequence reads were mapped to the reference database at species-level resolution. Image representing the comparison of relative abundance of the species in non-PMA and PMA treated samples during Flight 1 to Flight 3. **Figure S4**. Abundance of metagenomics reads related to the microbial phylum of PMA treated ISS environmental samples. **Figure S5**. Abundance of metagenomics reads related to the genus of PMA-treated ISS environmental samples. **Figure S6**. Abundance of antimicrobial resistance metagenomics reads in PMA- and non-PMA-treated samples from Flight 1, Flight 2, and Flight 3. **Figure S7.** Abundance of metagenomics reads associated with virulence. (ZIP 180 kb)

Additional file 4: **Table S4.** Virulence factors identified from the metagenomic sequences from all flight locations. (XLSX 27 kb)

Additional file 5: **Table S3.** Virulence genes of the *Pantoea* IF5SW-P1 strain identified in metagenome sequences of Flight 3. (XLSX 11 kb)

Additional file 6: **Table S5.** Functional analysis based on metagenomic reads for all locations and flights. (XLSX 21 kb)
