# Correction to: Succession and persistence of microbial communities and antimicrobial resistance genes associated with International Space Station environmental surfaces

**DOI:** 10.1186/s40168-018-0609-y

**Published:** 2018-12-04

**Authors:** Nitin Kumar Singh, Jason M. Wood, Fathi Karouia, Kasthuri Venkateswaran

**Affiliations:** 1 Jet Propulsion Laboratory, California Institute of Technology, 4800 Oak Grove Dr, Pasadena, CA 91109 USA; 2 Space Bioscience Division, NASA Ames Research Center, Moffett Field, CA USA; 3 Department of Pharmaceutical Chemistry, University of California San Francisco, San Francisco, CA USA

**Correction to: Microbiome (2018) 6:204**

10.1186/s40168-018-0585-2

Following publication of the original article [1], the authors reported a typographic error in scientific notation in the number of reads, the text should read as:

“Approximately 7.3 × 10^8^ reads associated with microorganisms were generated after high quality trimming from PMA (21 samples) and non-PMA treated (21 samples) samples. All metagenomics reads were normalized across all samples, which yielded 3.1 × 10^8^ in total, and 7.4 × 10^6^ assigned to each sample, without affecting the taxonomic diversity.”

The authors regret these errors and the inconvenience caused.
