# Supplementary material for: Succession and persistence of microbial communities and antimicrobial resistance genes associated with International Space Station environmental surfaces
Source: Microbiome. 2018 Nov 13;6:204. doi: 10.1186/s40168-018-0585-2 (PMC6234677; doi:10.1186/s40168-018-0585-2)
Supplement: Supplementary file 1 — Table S1. Initial DNA concentration and library yield for metagenomics analyses. (DOCX 16 kb) [file 40168_2018_585_MOESM1_ESM.docx]

**Supplementary Description S1: The characteristics of various locations of the International Space Station**

The International Space Station (ISS) is an orbital station that was been built by the United States (NASA) in collaboration with Russia (Roscosmos), Japan (JAXA), 11 nations of the European Space Agency (ESA), and Canada (CSA). The ISS is the largest and most complex international scientific project in history and is the largest human-made object to orbit Earth. The first ISS component, Russian Functional Cargo Block (FGB) also known as Zarya, was launched in 1998 and the last pressurized module, Permanent Multipurpose Module, was fitted in 2011 just prior to the retirement of the Space Shuttle Program. The ISS orbit can range from 278-460 km and is in an orbital inclination of 51.6°, which enables it to fly over 90% of Earth's population. The orbital speed is approximately 7.7 km/s (27,720 km/h) and as a result, it only takes 90 minutes to orbit around Earth. The ISS has a mass of 419 tons and a nominal pressurized volume of 916 m^3^. However, the habitable volume is only 388 m^3^, not including visiting vehicles. The pressurized module length is 73 m, whereas the truss length is 109 m and as such, the ISS can often be seen with the naked eye from Earth. The projected life of the ISS after completion of construction was about 10 years but funds have been provided to maintain the station and corresponding human spaceflight activities until 2024. During the current lifespan of the station over two hundred missions composed of periodic visits from international spacecraft for crew exchanges, resupply of food and other consumables, and many payloads for scientific investigations have occurred.

The Microbial Tracking-1 (MT-1) activities took place during crew increments 42 (11/10/14-3/11/15), 43 (3/11/15-6/11/15), and 47 (3/2/16-6/18/16). In particular, the sampling events occurred only when the Space Exploration Technologies (SpaceX) Dragon spacecraft was berthed to the ISS so samples could be returned to the ground for analysis within 4 weeks after being collected. All MT-1 related activities took place within the US on-orbit segments (USOS), i.e., Node 1, Node 2, Node 3, U.S. Laboratory module, and Permanent Multi-purpose Module (PMM).

Nodes are U.S. modules that connect the elements of the ISS. The first U.S. built element that was launched in Dec 1998 is Node 1 (Unity) and it connects the Russian and U.S. segments. In particular, the module provides six ports for berthing connections to the Zenith 1 Truss, U.S. Laboratory, Airlock for extravehicular activities (EVA), Node 3, FGB used primary for stowage and propulsion via the pressurized mating adapter (PMA) and visiting cargo vehicles. Essential ISS resources such as environmental control and life support systems, fluids, electrical and data systems are routed through Node 1 to supply other modules. Additionally, the module accommodates four racks for storage and a galley is a place of choice for sharing a meal.

Node 2 (Harmony) and Node 3 (Tranquility) were built by Europeans and are one rack bay longer than Node 1. They were launched into space in October 2007 and February 2010 respectively. Node 2 incorporates six berthing ports. It is attached to the forward end of the U.S. Laboratory and connects the European Laboratory, Columbus, on the starboard side, the Japanese laboratory, Kibo, on the port side, the PMA-2 and PMA-3 on the forward and zenith side respectively that provides docking locations for visiting vehicles. Whereas, on the nadir (Earth-facing) side, Node 2 provides a berthing port for cargo vehicles. The module is a utility hub, providing conversion and distribution of the electrical power, heating, cooling resources from the ISS Integrated Truss to the connected modules (U.S. Laboratory, Columbus, and Kibo) in addition to systems essential to support life, e.g., air and water, and support of the data and video exchange with ground and the rest of the ISS. Finally, Node 2 hosts crew quarters for four crew members. Node 3 is attached to the port side of Node 1, and the cupola is berthed on its nadir port. The PMM and the Bigelow Expandable Airlock Module (BEAM) are located on the forward and aft ports respectively. The module accommodates several ISS vital functions such as the air revitalization, oxygen generation, carbon dioxide removal and water recovery systems. It also contains elements for crew hygiene, the bathroom, and conditioning, treadmill and weight lifting device.

The U.S Laboratory module, Destiny, is the primary research laboratory for payloads that support a wild range of investigations addressing the effects of space environment, e.g., microgravity, on biological and physical processes to help sustain long-duration human spaceflight and exploration class missions in addition to the health, the safety, and quality of life for people all over the world. The module was launched in Feb 2001. The laboratory provides internal interfaces to accommodate the resource requirements of thirteen racks devoted to scientific research and eleven for fitting and control of ISS systems. Finally, the side that usually faces Earth has a large circular window (cupola) of very high optical quality for observatory purposes.

The Permanent Multipurpose Module (PMM), derived from the Leonardo Multipurpose Logistics Module which used to ferry cargo back and forth from the ISS, is an Italian build module berthed to the forward port of Node 3. The module was launch in February 2011. The primary function of the module is for stowage where the facility can host up to 16 racks containing a broad spectrum of equipment, experiments, and supplies. The module added 70 m^3^ of pressurized volume. Additional storage space can be found for bags in the aft end cone of the module. It added 2,472 additional cubic feet of pressurized volume for storage and for scientific use.

***Description of various locations sampled***

During MT-1 related activities samples (1 m^2^) were collected from eight different surface locations within Nodes 1-3, U.S. Laboratory, and PMM. Based on prioritization of surface locations and the efficiency of the crew procedure, the sampling plan was implemented as such: Node 3 (Locations #1-3), Node 1 (Locations #4 and 5), PMM (Location #6), U.S. Laboratory (Location #7), and Node 2 (Locations #8 and control).

**Node 3:**

**Location #1: Port Panel of the cupola**

The cupola is a small module devoted to the observation of operations outside the ISS such as robotic activities, the approach of vehicles, and EVA. The module is berthed to the nadir port of Node 3. It was built in Europe by the Italian Space Agency and was launched in February 2010. The module has six side windows and a center window facing Earth, all of which are fused silica and borosilicate glass panes, window heaters, and thermistors equipped with shutters to protect them from contamination and collision with natural or manmade orbital debris. The cupola can accommodate two crewmembers simultaneously and is designed to house the robotic workstation that controls the ISS’s remote manipulator arm. Additionally, it offers spectacular views of Earth and celestial bodies and is a popular spot for crewmembers during downtime. The panel (port side) on the way to the cupola was sampled. The panel is made of aluminum with a polyurethane topcoat (e.g., Aeroglaze A276 or BMS10-60).

**Location #2: Forward side panel wall of the Waste and Hygiene Compartment (WHC)**

The Waste and Hygienic Compartment (WHC), the space toilet, was the second toilet facility to arrive on the Station, in November 2008 to accommodate the doubling of crewmembers onboard the ISS. Initially installed in the U.S. Laboratory, the booth-like compartment is a Russian-built toilet system outfitted with extra sensors to help automate flush tank fill and is now in Node 3. The collection and retention of liquid and solid waste are directed by airflow. The unit separately channels liquid and solid waste. While the solid waste goes to a holding tank, the liquid is recycled as part of the regenerative Environmental Control and Life Support Systems (ECLSS). In particular, elements within ECLSS such as the urine and water processor assemblies and the portable water dispenser are designed to provide drinking-quality water through the reclamation of wastewater including urine and hygiene wastes. The water produced is used by the crew and for work onboard the ISS. The wall surface on the back of the WHC was sampled. The panel (forward side) is made of aluminum with a polyurethane topcoat (e.g., Aeroglaze A276 or BMS10-60).

**Location #3: The foot platform of the Advanced Resistive Exercise Device (ARED)**

The Advanced Resistive Exercise Device (ARED) functions to maintain crew health in space. Crew members exercise daily on ARED to maintain their preflight muscle and bone strength and endurance. The ARED was taken to the ISS on space shuttle mission STS-126 in November 2008 and put into service in January 2009. The system is based on a piston and flywheel to simulate free-weight exercises in normal gravity to work for all major muscle groups through squats, deadlifts, and calf raises. The primary goal of the ARED is to maintain muscle strength and mass, resistive exercise also helps astronauts increase endurance for physically demanding tasks such as EVAs. The service life of the system is at least fifteen years with the assumption of 80% of use will be performed using the lift bar assembly and 20% using the cable assembly. The foot platform of the ARED was sampled. The platform is made of gold anodized aluminum and was sampled during F1. However, the platform sampled during F2 and F3 was subsequently covered with a black no-slip laminate (mineral grit embedded in an adhesive paper).

**Node 1:**

**Location #4: Surface of the dining table**

The original dining table was a squared surface (~0.6 m^2^) located in the galley of Node 1. Neither the US nor the Russian agencies have an official record of the existence of this table. It was custom made by the first ISS permanent crewmembers from the material used to protect the Russian oxygen generator candles. The table had two handrails and tapes, hook and loop enclosures, and clips were added so items could be strapped to the table. The main function of the table was for dining however, at many instances the crewmembers were also using the table to strap tools and other work-related items. As the number of permanent crewmembers increased over time, the table was not large enough to accommodate all the astronauts to share a meal. Therefore an initiative to build a new dining table took place in 2014 through the OB Vehicle Integration Office in collaboration with a high school to design and build a new dining table that is large enough to accommodate six crewmembers and strong enough to resist kicks and pushes. The table is rectangular when fully open (~1.25 m^2^). The table is composed of a large and small leaf with latches in the middle and handrails on each side. The table was launched onboard Orbital 6 and arrived at the ISS in March 2016. The crew added tape, hook and loop enclosures, clips, and bungees to the table to hold their utensils and food in place. If any of these restraints gets dirty or stop working, they can be thrown away and replaced. The material for the original dining table is uncertain but could be based on polyimide. During F1 and F2, samples were collected from the original table whereas F3 was done on the new dining table just a few weeks after the table arrived onboard the ISS. The new table is made of aluminum and stainless steel. The surfaces were anodized and passivated to give the desired color and provide a hard surface coating to inhibit corrosion.

**Location #5: Overhead 4- Zero-G Stowage Rack**

Zero-G Stowage Racks (ZSRs) are fabric racks that are used onboard the ISS to provide stowage accommodations. The ZSR is a lightweight, on-orbit stowage restraint system. The ZSR comprises two elements: a collapsible shell and a fabric insert. The shell is an aluminum frame that provides a standardized interface to the insert. The fabric inserts can be configured to carry sub-containers that include the stowage trays mentioned above, as well as various types and sizes of soft-sided cargo bags. The 1.2m³ (42.8 ft³) ZSR is not designed to transport cargo during launch and landing phases. The front panel of the overhead 4 ZSR was sampled. The white fabric surface material is based on Nomex. The content of the rack changed over time. During F1, the rack contained the battery pantry, Printer Cartridges, Office supplies (tape, Ziploc, pens), AC Dry Vacuum Supplies, Cameras and cables, and Trash bags. Whereas during F2 the rack contained camera mounts, cables, blankets, Ziplock bags, and labels. Finally, during Flight 3, miscellaneous EVA camera parts were stow in the rack.

**The Permanent Multi-purpose Module:**

**Location #6: Port 1- Zero-G Stowage Rack and Port-2 Rack wall**

As previously described, the Zero-G Stowage Racks (ZSRs) are fabric racks that are used onboard the ISS to provide stowage accommodations. The front surface of the port 1 ZGR was sampled during Flight 1 and Flight 2. The white fabric surface material is based on Nomex. During Flight 1, the rack contained clothes, Crew Preference items, Office Supplies, Small Tools (Leatherman, flashlight), and ISS Medical Accessory Kit (IMAK). Whereas during Flight 2 the rack contained clothes, Hygiene Towels, Cables, Jumpers, Caps, and Food. During Flight 3, the PMM configuration changed and Port-1 ZSG was not accessible for sampling. Therefore, the Port-2 panel was sampled instead. It is composed of aluminum honeycomb. Near the sampling location, miscellaneous cables and accessories were present.

**The U.S. Laboratory:**

**Location #7: Overhead 3 panel surface**

The Materials Science Research Rack-1 (MSRR-1) is being used for basic materials research in the microgravity environment of the ISS. MSRR-1 can accommodate and support diverse Experiment Modules (EMs). In this way, many material types, such as metals, alloys, polymers, semiconductors, ceramics, crystals, and glasses, are studied to discover new applications for existing materials and new or improved materials. The overhead3 space area (LAB103) was sampled. The panel is made of aluminum with a polyurethane topcoat (e.g., Aeroglaze A276 or BMS10-60).

**Node 2:**

**Location #8: Crew Quarters-2 Bump-out exterior aft wall**

The Crew Quarters (CQ) is a permanent personal space for crew members to sleep, perform personal recreation and communication, as well as provide on-orbit stowage of personal belongings. The CQ was designed to provide 2.1 m^3^ of interior volume with an individual ventilation system, acoustical mitigation materials, radiation protection, light, and connections for laptop and internet. The CQs provide visual, light, and acoustic isolation for the crew member. The CQ was deployed in 2008 and over a 2-year period, four CQs were launched to the ISS and currently reside in Node 2. The crew also uses the CQ for performing tasks such as donning/doffing clothing and some minimal personal hygiene. The structure of the CQ can be divided into three main areas: bump-out, rack, and pop-up. To maximize the amount of interior volume, bump-out and pop-up were designed to contain key features. The crew quarter-2 Bump-out exterior aft wall was sampled during MT-1. The bump-out houses the ventilation system and is comprised of aluminum Panels covered in acoustic absorption blankets which consists of a quilted configuration of Gore-Tex®, BISCO®, Durette felt, and Nomex. Due to the accumulation of dust and debris, the crew is tasked to clean the ventilation system every 9 months. Additionally, weekly vacuum cleaning of the exterior mesh screens takes place to reduce dust and debris build-up and provides crew members a safe sleeping environment.
