# Supplementary figures and images for: Succession and persistence of microbial communities and antimicrobial resistance genes associated with International Space Station environmental surfaces
Source: Microbiome. 2018 Nov 13;6:204. doi: 10.1186/s40168-018-0585-2 (PMC6234677; doi:10.1186/s40168-018-0585-2)

## PMA

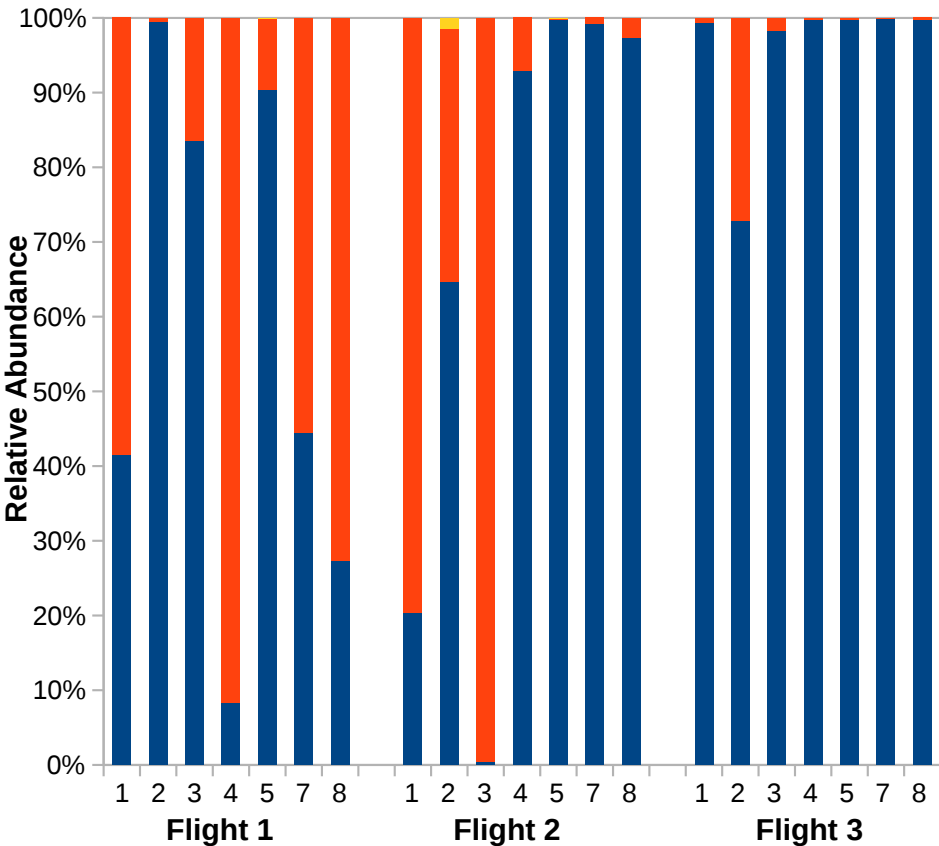

## No PMA

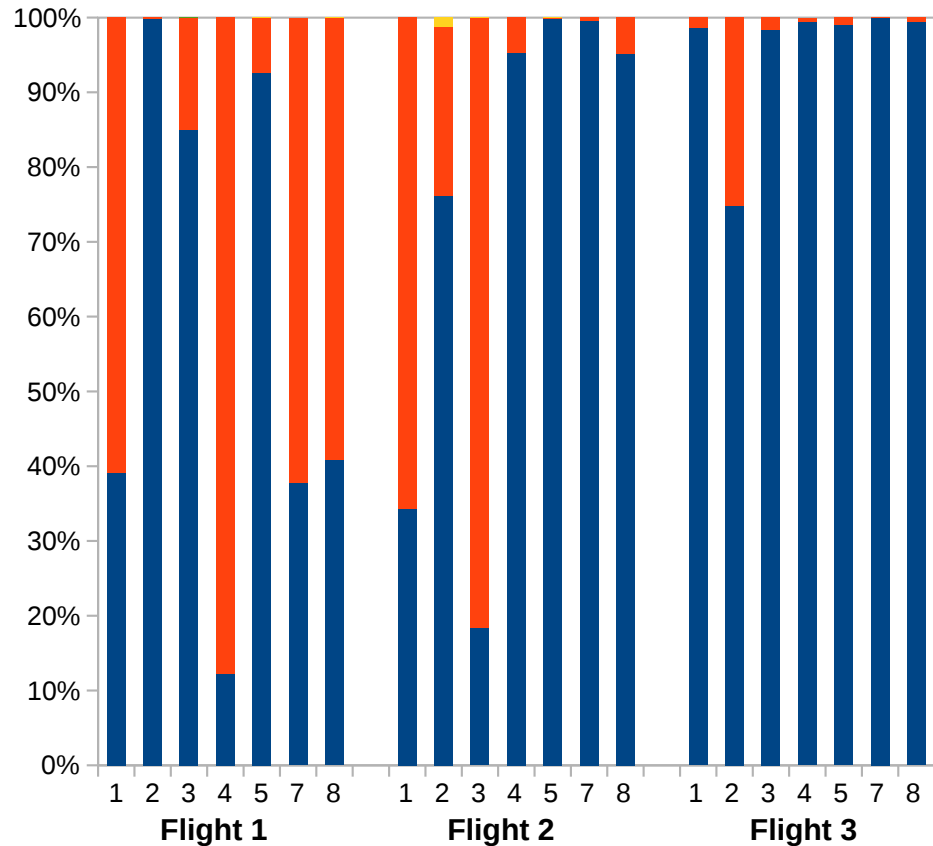

■ Bacteria - 74%

■ Eukarya - 25%

■ Archaea - <1%

■ Virus - <1%

Supplement: Supplementary file 3 — Figure S1. Percentage of total reads obtained from PMA and non-PMA treated samples. Figure S2. Proportional abundance at flight level. The proportional abundance of different flight sampling shows the presence of all domains (Bacteria, Archaea, and Eukaryota). There is a noticeable increase in bacterial population from Flight 1 to Flight 3. Figure S3. Top 25 species dominating the microbial composition of ISS samples. Normalized sequence reads were mapped to the reference database at species-level resolution. Image representing the comparison of relative abundance of the species in non-PMA and PMA treated samples during Flight 1 to Flight 3. Figure S4. Abundance of metagenomics reads related to the microbial phylum of PMA treated ISS environmental samples. Figure S5. Abundance of metagenomics reads related to the genus of PMA-treated ISS environmental samples. Figure S6. Abundance of antimicrobial resistance metagenomics reads in PMA- and non-PMA-treated samples from Flight 1, Flight 2, and Flight 3. Figure S7. Abundance of metagenomics reads associated with virulence. (ZIP 180 kb) [file 40168_2018_585_MOESM3_ESM.zip › MBIO-D-18-00304_figure-s1.pdf]

## Propotional Abundance

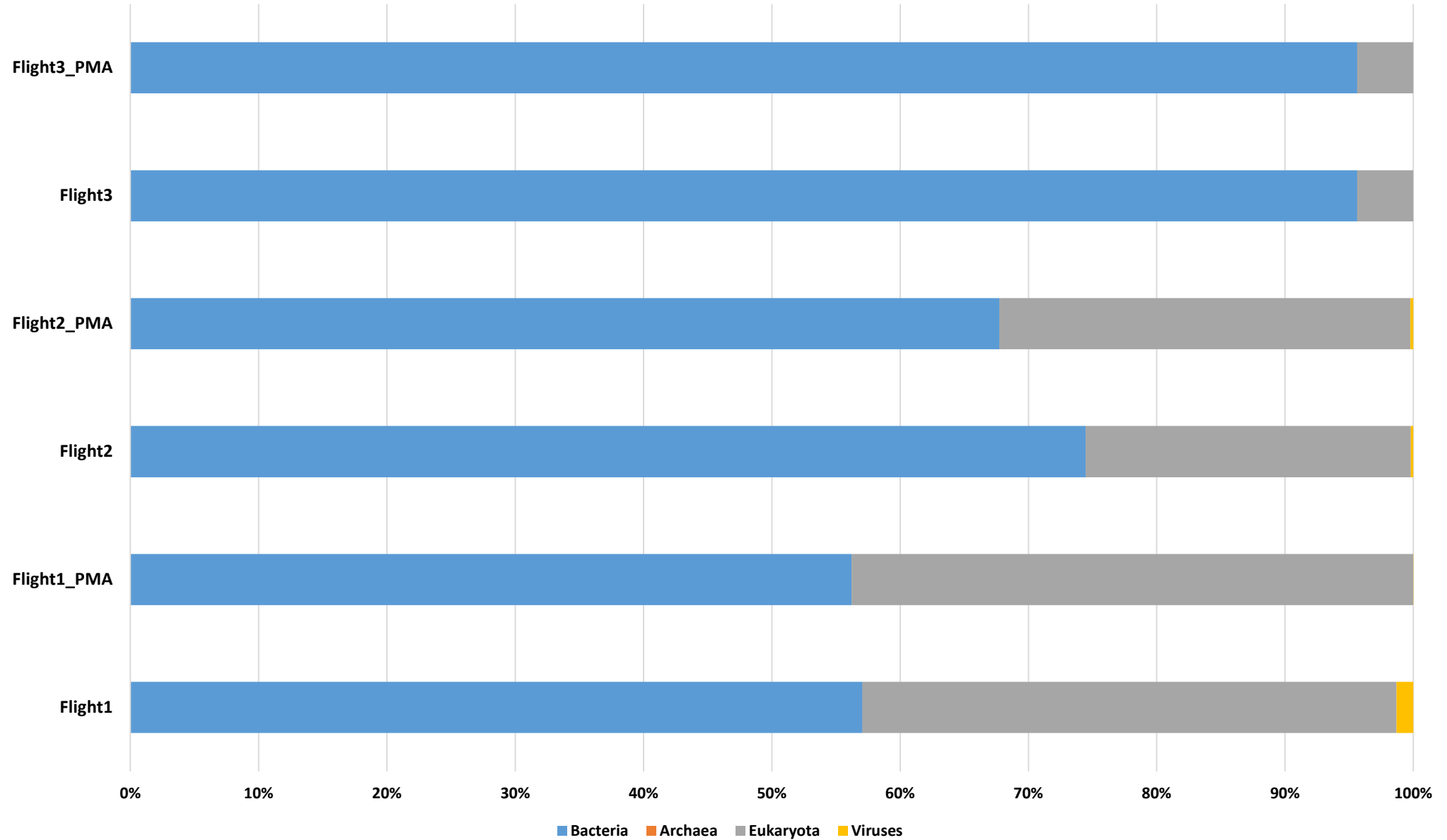

Supplement: Supplementary file 3 — Figure S1. Percentage of total reads obtained from PMA and non-PMA treated samples. Figure S2. Proportional abundance at flight level. The proportional abundance of different flight sampling shows the presence of all domains (Bacteria, Archaea, and Eukaryota). There is a noticeable increase in bacterial population from Flight 1 to Flight 3. Figure S3. Top 25 species dominating the microbial composition of ISS samples. Normalized sequence reads were mapped to the reference database at species-level resolution. Image representing the comparison of relative abundance of the species in non-PMA and PMA treated samples during Flight 1 to Flight 3. Figure S4. Abundance of metagenomics reads related to the microbial phylum of PMA treated ISS environmental samples. Figure S5. Abundance of metagenomics reads related to the genus of PMA-treated ISS environmental samples. Figure S6. Abundance of antimicrobial resistance metagenomics reads in PMA- and non-PMA-treated samples from Flight 1, Flight 2, and Flight 3. Figure S7. Abundance of metagenomics reads associated with virulence. (ZIP 180 kb) [file 40168_2018_585_MOESM3_ESM.zip › MBIO-D-18-00304_figure-s2.pdf]

No PMA

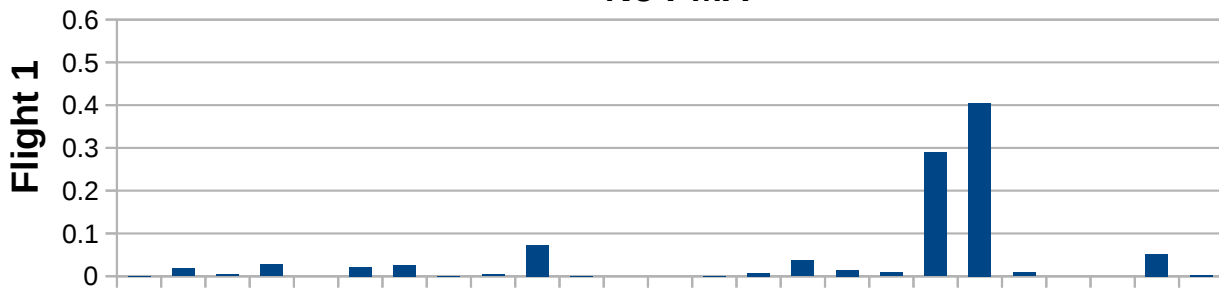

PMA

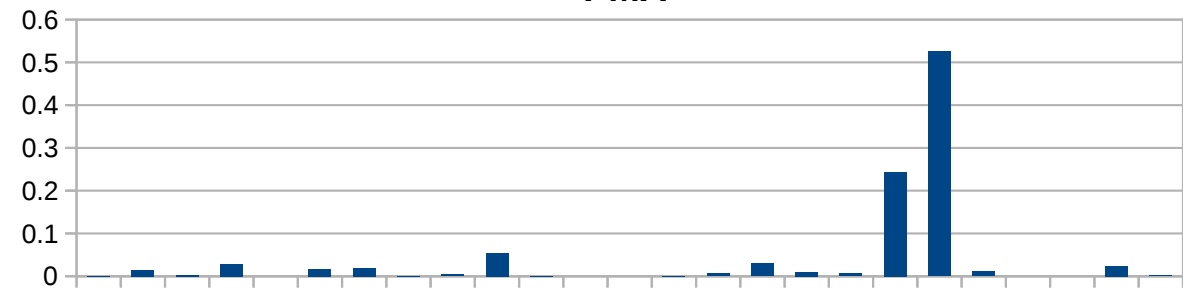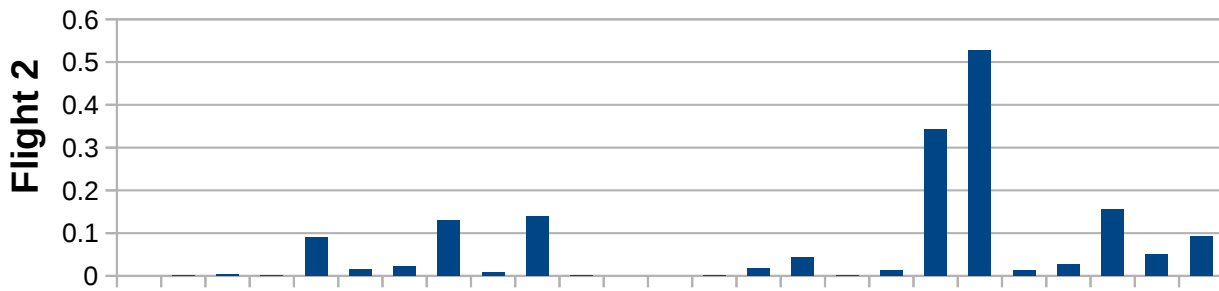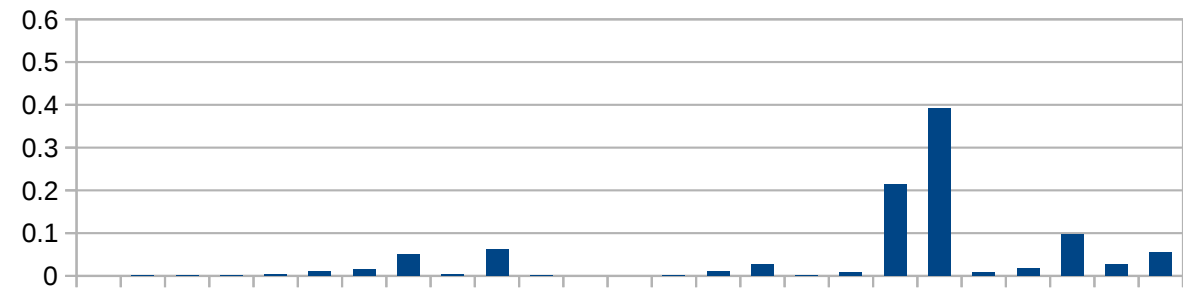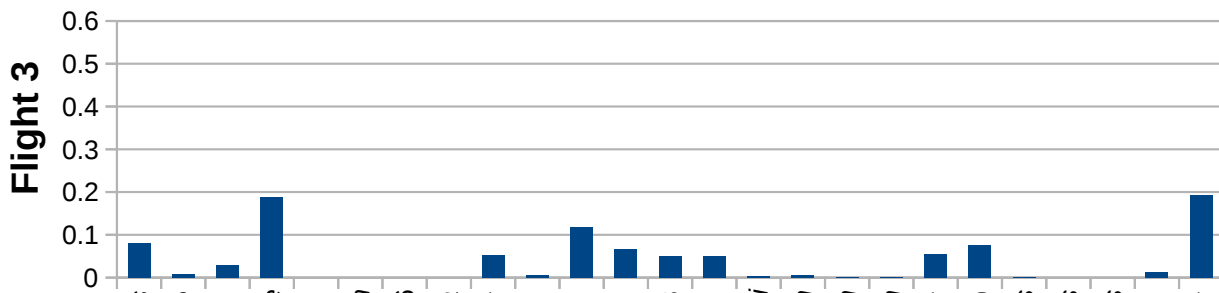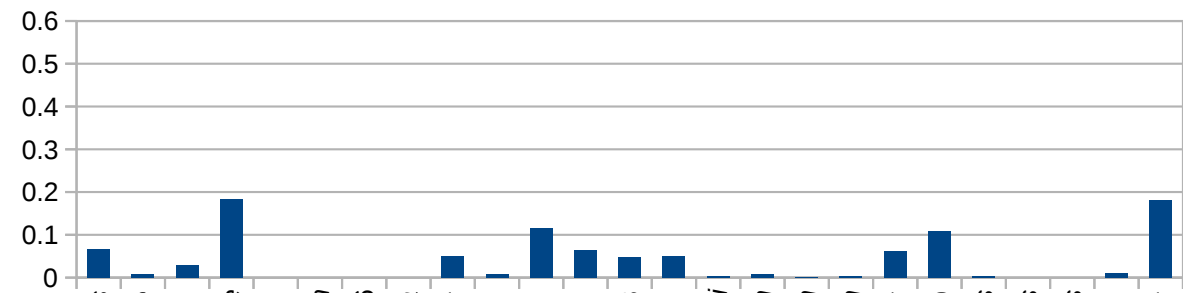

Supplement: Supplementary file 3 — Figure S1. Percentage of total reads obtained from PMA and non-PMA treated samples. Figure S2. Proportional abundance at flight level. The proportional abundance of different flight sampling shows the presence of all domains (Bacteria, Archaea, and Eukaryota). There is a noticeable increase in bacterial population from Flight 1 to Flight 3. Figure S3. Top 25 species dominating the microbial composition of ISS samples. Normalized sequence reads were mapped to the reference database at species-level resolution. Image representing the comparison of relative abundance of the species in non-PMA and PMA treated samples during Flight 1 to Flight 3. Figure S4. Abundance of metagenomics reads related to the microbial phylum of PMA treated ISS environmental samples. Figure S5. Abundance of metagenomics reads related to the genus of PMA-treated ISS environmental samples. Figure S6. Abundance of antimicrobial resistance metagenomics reads in PMA- and non-PMA-treated samples from Flight 1, Flight 2, and Flight 3. Figure S7. Abundance of metagenomics reads associated with virulence. (ZIP 180 kb) [file 40168_2018_585_MOESM3_ESM.zip › MBIO-D-18-00304_figure-s3.pdf]

**A**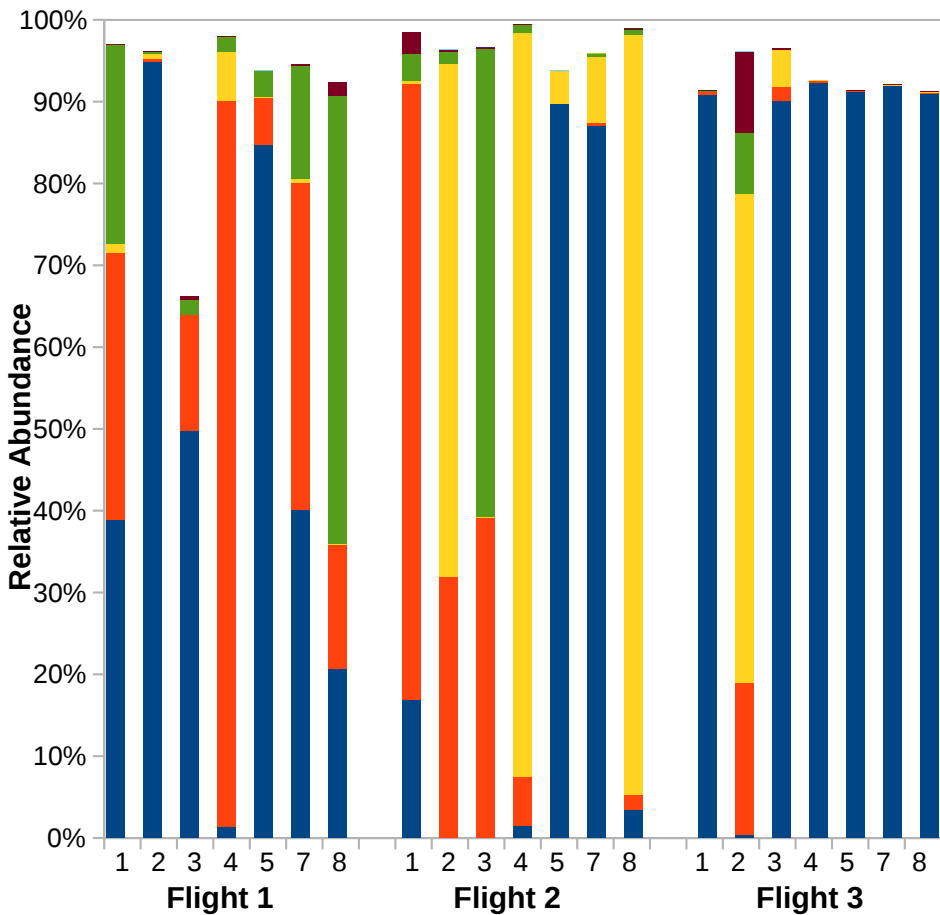**B**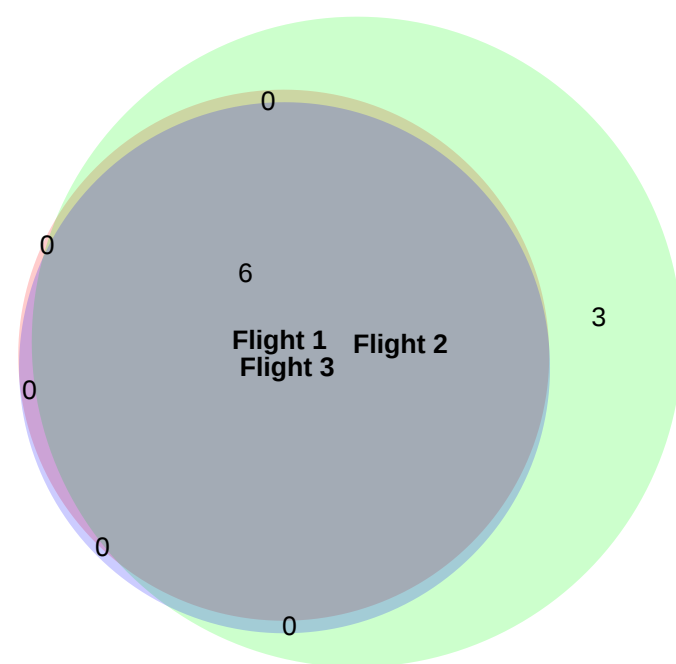**C**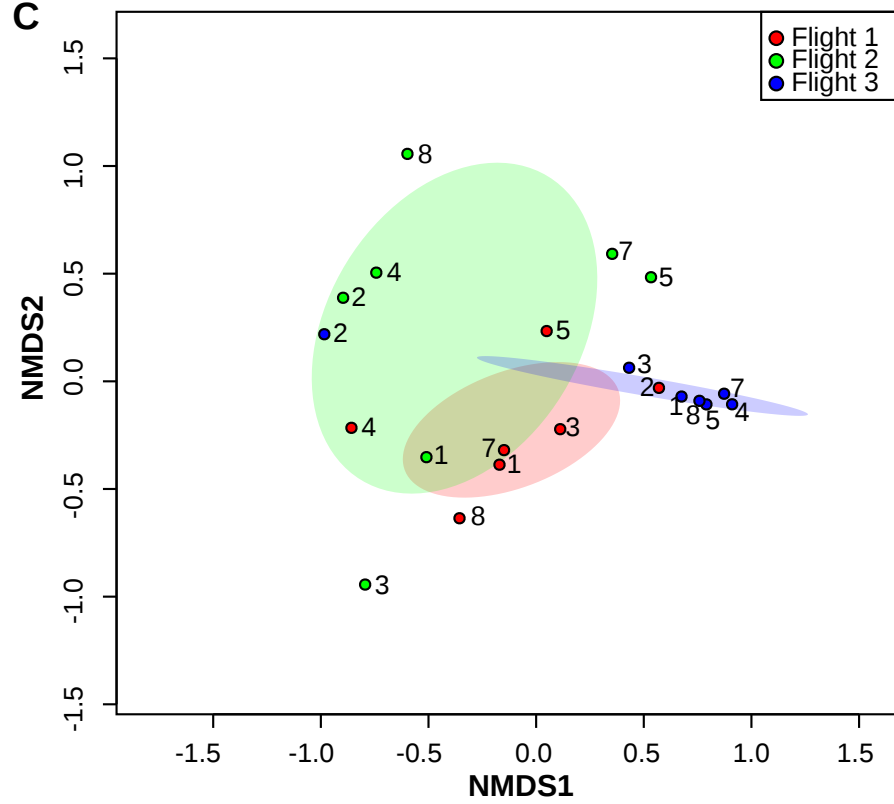

Supplement: Supplementary file 3 — Figure S1. Percentage of total reads obtained from PMA and non-PMA treated samples. Figure S2. Proportional abundance at flight level. The proportional abundance of different flight sampling shows the presence of all domains (Bacteria, Archaea, and Eukaryota). There is a noticeable increase in bacterial population from Flight 1 to Flight 3. Figure S3. Top 25 species dominating the microbial composition of ISS samples. Normalized sequence reads were mapped to the reference database at species-level resolution. Image representing the comparison of relative abundance of the species in non-PMA and PMA treated samples during Flight 1 to Flight 3. Figure S4. Abundance of metagenomics reads related to the microbial phylum of PMA treated ISS environmental samples. Figure S5. Abundance of metagenomics reads related to the genus of PMA-treated ISS environmental samples. Figure S6. Abundance of antimicrobial resistance metagenomics reads in PMA- and non-PMA-treated samples from Flight 1, Flight 2, and Flight 3. Figure S7. Abundance of metagenomics reads associated with virulence. (ZIP 180 kb) [file 40168_2018_585_MOESM3_ESM.zip › MBIO-D-18-00304_figure-s4.pdf]

**A**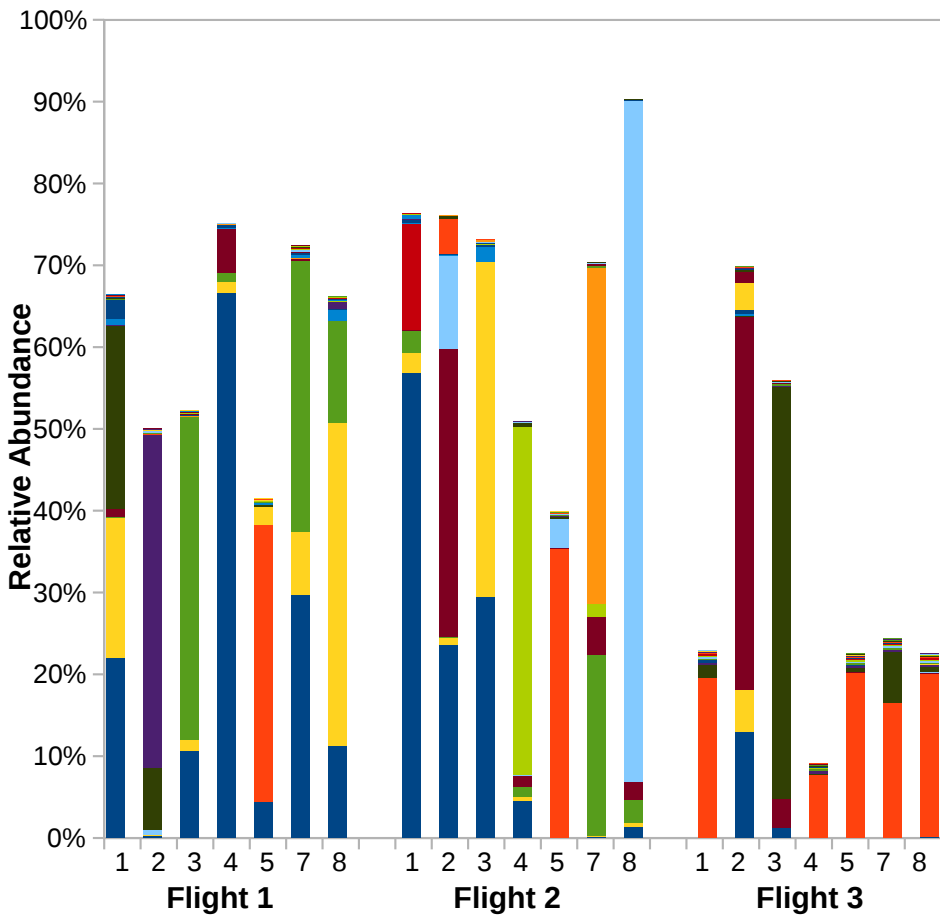**B**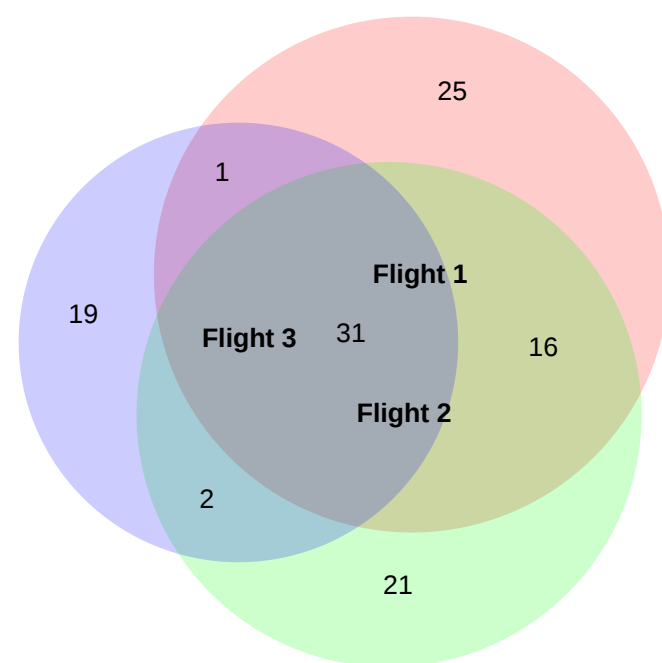**C**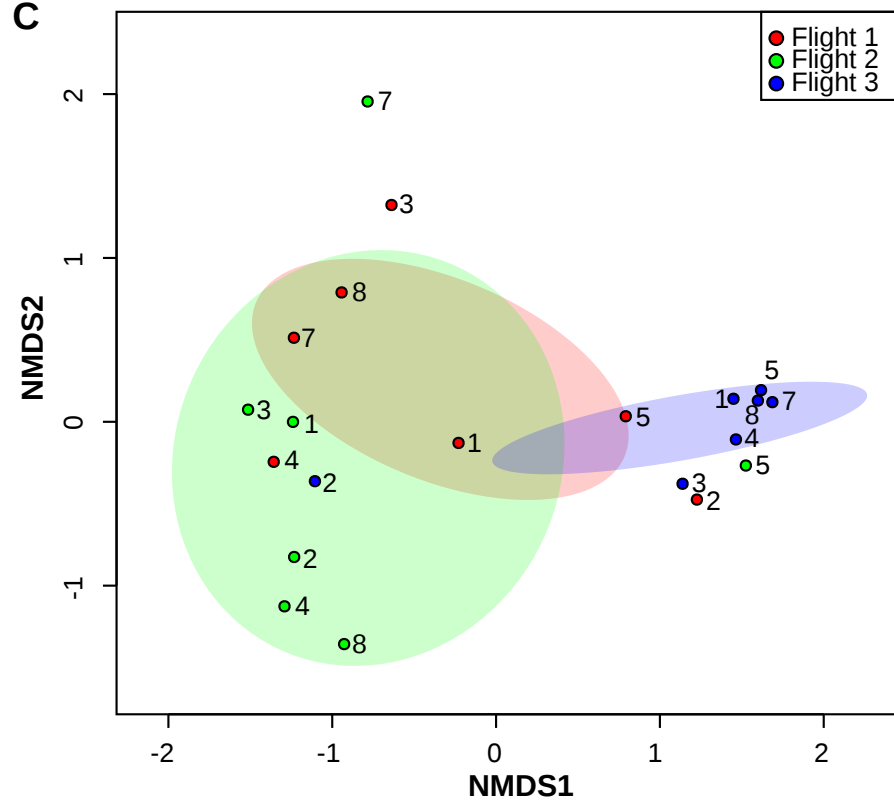

Supplement: Supplementary file 3 — Figure S1. Percentage of total reads obtained from PMA and non-PMA treated samples. Figure S2. Proportional abundance at flight level. The proportional abundance of different flight sampling shows the presence of all domains (Bacteria, Archaea, and Eukaryota). There is a noticeable increase in bacterial population from Flight 1 to Flight 3. Figure S3. Top 25 species dominating the microbial composition of ISS samples. Normalized sequence reads were mapped to the reference database at species-level resolution. Image representing the comparison of relative abundance of the species in non-PMA and PMA treated samples during Flight 1 to Flight 3. Figure S4. Abundance of metagenomics reads related to the microbial phylum of PMA treated ISS environmental samples. Figure S5. Abundance of metagenomics reads related to the genus of PMA-treated ISS environmental samples. Figure S6. Abundance of antimicrobial resistance metagenomics reads in PMA- and non-PMA-treated samples from Flight 1, Flight 2, and Flight 3. Figure S7. Abundance of metagenomics reads associated with virulence. (ZIP 180 kb) [file 40168_2018_585_MOESM3_ESM.zip › MBIO-D-18-00304_figure-s5.pdf]

# Antimicrobial resistance

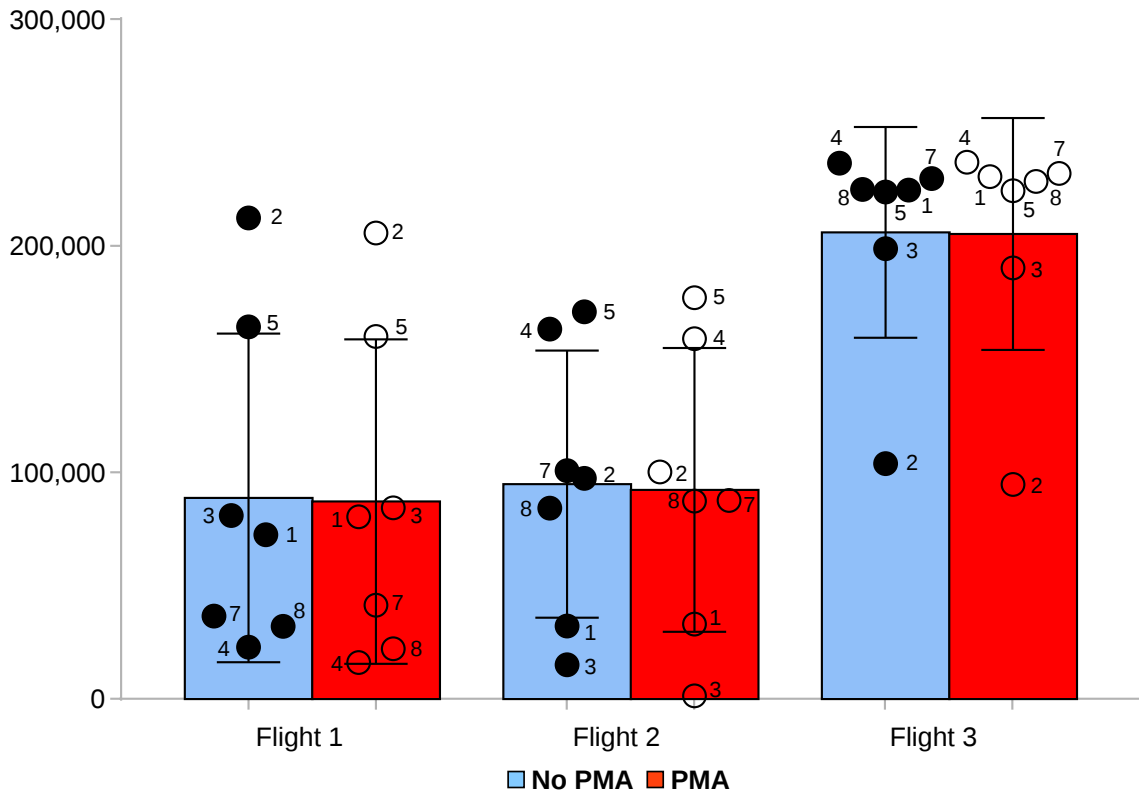

Supplement: Supplementary file 3 — Figure S1. Percentage of total reads obtained from PMA and non-PMA treated samples. Figure S2. Proportional abundance at flight level. The proportional abundance of different flight sampling shows the presence of all domains (Bacteria, Archaea, and Eukaryota). There is a noticeable increase in bacterial population from Flight 1 to Flight 3. Figure S3. Top 25 species dominating the microbial composition of ISS samples. Normalized sequence reads were mapped to the reference database at species-level resolution. Image representing the comparison of relative abundance of the species in non-PMA and PMA treated samples during Flight 1 to Flight 3. Figure S4. Abundance of metagenomics reads related to the microbial phylum of PMA treated ISS environmental samples. Figure S5. Abundance of metagenomics reads related to the genus of PMA-treated ISS environmental samples. Figure S6. Abundance of antimicrobial resistance metagenomics reads in PMA- and non-PMA-treated samples from Flight 1, Flight 2, and Flight 3. Figure S7. Abundance of metagenomics reads associated with virulence. (ZIP 180 kb) [file 40168_2018_585_MOESM3_ESM.zip › MBIO-D-18-00304_figure-s6.pdf]

# Virulence

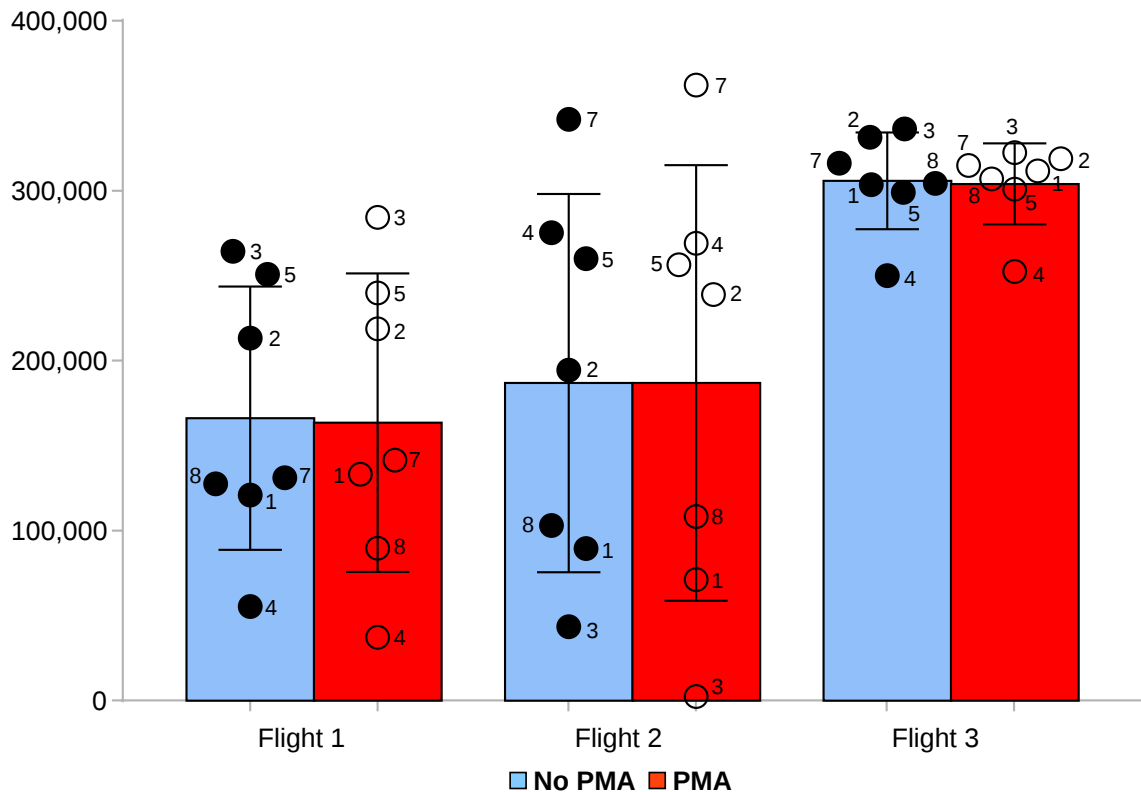

Supplement: Supplementary file 3 — Figure S1. Percentage of total reads obtained from PMA and non-PMA treated samples. Figure S2. Proportional abundance at flight level. The proportional abundance of different flight sampling shows the presence of all domains (Bacteria, Archaea, and Eukaryota). There is a noticeable increase in bacterial population from Flight 1 to Flight 3. Figure S3. Top 25 species dominating the microbial composition of ISS samples. Normalized sequence reads were mapped to the reference database at species-level resolution. Image representing the comparison of relative abundance of the species in non-PMA and PMA treated samples during Flight 1 to Flight 3. Figure S4. Abundance of metagenomics reads related to the microbial phylum of PMA treated ISS environmental samples. Figure S5. Abundance of metagenomics reads related to the genus of PMA-treated ISS environmental samples. Figure S6. Abundance of antimicrobial resistance metagenomics reads in PMA- and non-PMA-treated samples from Flight 1, Flight 2, and Flight 3. Figure S7. Abundance of metagenomics reads associated with virulence. (ZIP 180 kb) [file 40168_2018_585_MOESM3_ESM.zip › MBIO-D-18-00304_figure-s7.pdf]
